# A viral protein disrupts rice cell wall integrity and modulates interactions with viruses and insects

**DOI:** 10.1007/s44154-026-00287-4

**Published:** 2026-02-19

**Authors:** Jialin Zhang, Wenyang Ye, Shanshan Li, Chaoyi Dong, Fanrong Peng, Ci Wang, Jiaqi Qin, Qun Hu, Jie Zhang, Jianguo Wu, Shanshan Zhao

**Affiliations:** 1 State Key Laboratory of Agriculture and Forestry Biosecurity, Center for Genetic Improvement, College of Plant Protection, Vector-Borne Virus Research Center, Fujian Agriculture and Forestry University, Fuzhou, 350002 China; 2 Institute of Biotechnology and Germplasm Resources, Yunnan Provincial Key Lab of Agricultural Biotechnology, Yunnan Academy of Agricultural Sciences, Kunming, 650205 China

**Keywords:** Rice grassy stunt virus, p2, Cell wall, Pathogenesis

## Abstract

The plant cell wall provides structural support and serves as a barrier against pathogen invasion. Rice grassy stunt virus (RGSV) infection suppresses genes involved in cell wall biosynthesis, but the underlying mechanism remains unclear. To further investigate this phenomenon, we generated transgenic rice lines overexpressing the RGSV-encoded p2 protein. These transgenic lines exhibited a brittle phenotype with reduced plant height, thinner sclerenchyma cell walls, decreased cellulose and increased lignin contents. Biochemical and microscopic analyses confirmed that mechanical strength of the cell wall was significantly weakened in p2-expressing plants. Notably, immunoblotting and in situ hybridization revealed partial localization of p2 to the cell wall, suggesting potential structural association. Transcriptome analysis revealed that p2 expression significantly altered the expression of genes involved in cell wall organization, hormone signaling, and pathogen interactions, suggesting a mechanistic basis for the observed phenotypes. Additionally, p2 transgenic lines exhibited increased susceptibility to multiple viruses, but unexpectedly showed enhanced resistance to the brown planthopper (BPH, *Nilaparvata lugens*), a major phloem-feeding pest. These findings reveal that a single viral protein can remodel the cell wall to influence both pathogen susceptibility and insect resistance, highlighting the broader ecological impacts of virus-induced cell wall remodeling in plants.

**Supplementary Information:**

The online version contains supplementary material available at 10.1007/s44154-026-00287-4.

## Introduction

Plant cell wall is a thin, strong and pliable extracellular layer with unique properties and functions (Cosgrove 2005; Hofte and Voxeur 2017). It is mainly composed of highly integrated and structurally complex polysaccharides, including cellulose, hemicellulose and pectin (Cosgrove 2015). Cell wall can not only strengthen plant body, but also play key roles related to plant growth and responses to environmental cues (Malinovsky et al. 2014; Le Gall et al. 2015; Kesten et al. 2017). The antagonistic host–pathogen relationship at the walls are great interests to plant researchers in many laboratories (Hematy et al. 2009; Bacete et al. 2018). It is well known that although plant cell wall can serve as physical barriers to prevent pathogen invasions, many pathogens have also evolved to produce cell wall modification enzymes to allow them to penetrate the walls and to gain access to nutrients in protoplasts (Reem et al. 2016; Wang et al. 2019). For example, the reduction of expansin content in walls can alter wall structures to prevent pathogen penetrations (Ding et al. 2008; Domingo et al. 2009). Silencing the expression of *GLYCEROL-3-PHOSPHATE ACYLTRANSFERASE 6* (*GPAT6*) gene in tomato and *Nicotiana benthamiana* leaves changes the outer wall structure and increases plant susceptibility to pathogen infections (Fawke et al. 2019). Because plant viruses cannot be transmitted to plants through gentle contact with plant surface, the role of cell wall during virus invasion is largely unknown (Chen et al. 2000; Boevink and Oparka 2005).

Rice (*Oryza sativa*) is an ideal model plant for the studies on the relationship between virus and cell walls (Shimizu et al. 2007; Satoh et al. 2011, 2013; Zhang and Zhou 2011; Budot et al. 2014; Qin et al. 2019). The height and mechanical strength of rice plants are strongly influenced by cell walls and are important agronomic traits associated with lodging and stress resistance (Tayi et al. 2016; Hu et al. 2017). Plant stunting is a common disease symptom in rice plants infected with viruses, including rice stripe virus (RSV) (Wu et al. 2015, 2017; Yao et al. 2019), rice grassy stunt virus (RGSV) (Arif et al. 2018; Sui et al. 2018), rice dwarf virus (RDV) (Jin et al. 2016; Zhao et al. 2017), Southern rice black-streaked dwarf virus (SRBSDV) (Xu and Zhou 2017), rice ragged stunt virus (RRSV) (Zhang et al. 2016), rice black-streaked dwarf virus (RBSDV) (He et al. 2017; Xie et al. 2018), and rice tungro spherical virus (RTSV) (Budot et al. 2014; Macovei et al. 2018). Many genome-wide gene expression studies have revealed that the expressions of many cell wall biosynthesis-related genes are suppressed during RDV or RSV infection in rice plants (Shimizu et al. 2007; Satoh et al. 2010, 2011), implying that the stunting phenotype may be controlled by the genes associated with plant morphology and development. Interestingly, the expressions of several genes encoding cellulose synthases and arabinogalactan proteins important for cell wall formations have also been found to be substantially suppressed during RGSV or RTSV infection in rice (Satoh et al. 2013; Budot et al. 2014). To date, the mechanism(s) underlying the effects of virus infection on rice cell wall biosynthesis remain poorly understood.

RGSV is a member in the genus *Tenuivirus*, family *Phenuiviridae*, and is one of the most widespread rice viruses that cause severe diseases in rice fields in several Southeast Asian countries (Shikata et al. 1980; Hibino 1996). RGSV is transmitted by brown planthopper (BPH; *Nilaparvata lugens*) in a persistent-propagative manner (Hibino 1996). Infection of RGSV in rice causes strong plant stunting, excessive tillering, and leaf chlorosis (Shikata et al. 1980). RGSV genome comprises six single-stranded RNA segments, known as RNA1 to 6 based on the size of the segments (from the largest to the smallest size). All the six RNA segments shear an almost identical 5' terminal 17 nucleotide sequence and employ an ambisense coding strategy for 12 open reading frames (Ramirez 2008). To date, four RGSV-encoded proteins have been functionally characterized. Specifically, the pC1 protein encoded by the negative sense-strand RNA1 is an RNA-dependent RNA polymerase (RdRP) (Toriyama et al. 1997), the p5 protein encoded by the viral sense-strand RNA5 is a viral suppressor of RNA silencing (VSR) (Zhang et al. 2015), the pC5 protein encoded by the complementary sense-strand RNA5 is a capsid protein and a major component of thin filamentous particles (Toriyama et al. 1997), and the pC6 protein encoded by the virion sense-strand RNA6 is a nonstructural protein important for virus movement (Hiraguri et al. 2011).

Although RGSV infection in rice induces plant stunting, a common disease symptom in virus-infected plants, it was unclear which and how RGSV protein modulates this symptom. Ours group recently found that RGSV encoded P3 protein causes dwarfing and excess tillering phenotype through inducing a E3 ligase to target OsNRPD1 for degradation (Xu et al. 2020; Zhang et al. 2020; Wu et al. 2025). In this study, we investigate the function of the RGSV-encoded p2 protein. We show that p2 overexpression disrupts cellulose synthesis and alters cell wall integrity, leading to a brittle phenotype and increased virus susceptibility. Intriguingly, this same modification confers increased resistance to brown planthopper feeding. This finding indicates that a single RGSV protein can manipulate cell wall biosynthesis pathway to enable an efficient virus infection. The results presented in this paper should advance our knowledge on the functions of RGSV proteins and on the mechanism(s) controlling the onset of a common virus symptom in the infected rice plants.

## Results

### Overexpression of RGSV p2 impairs rice development and compromises mechanical strength

This study aimed to elucidate the role of the RGSV‑encoded p2 protein during viral infection in rice. The full‑length RNA2 sequence of RGSV was cloned and introduced into the rice cultivar Nipponbare to generate p2 overexpression (p2 OE) transgenic lines. Three independent p2 OE lines (p2 OE#1, p2 OE#2, and p2 OE#3) were selected for further analysis. Compared with wild‑type (WT) plants, all three transgenic lines exhibited reduced plant height, fewer productive panicles, and lower 1,000‑grain weight (Fig. S1). To further dissect the phenotypic changes associated with p2 expression, we investigated internode length and cell morphology. The uppermost (IV) and second‑uppermost (III) internodes in p2 OE plants were significantly shorter than those in WT plants (Fig. S2A and B). Microscopic analysis of leaf sheath cells revealed significantly reduced cell length in transgenic plants, suggesting that p2 expression suppresses cell expansion during rice development (Fig. S2C and D).

**Fig. 1:**
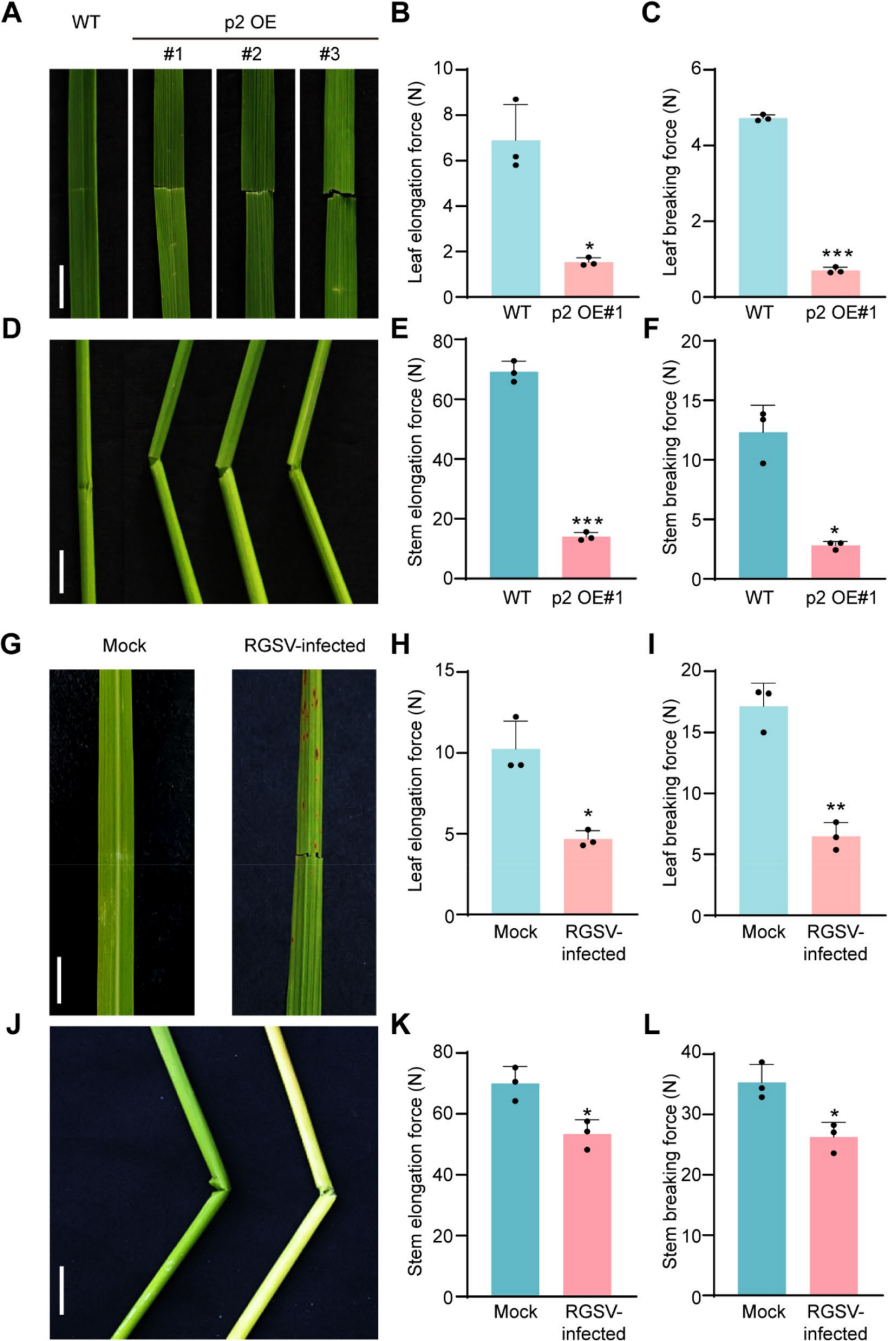
Growth phenotypes and the mechanical strength of the p2 OE transgenic lines and RGSV-infected rice plants. **A**&**D** An easily broken flag leaf (**A**) and culm (**D**) of the three p2 OE line compared with WT plant in the mature stage. Scale bar, 3 cm. **B**&**E** The elongation force of leaf (**B**) and second upper internode (**E**) of the three p2 OE line plants compared with WT plant. Scale bar, 3 cm. Data were shown as mean ± SD (*n* = 3). **C**&**F** The breaking force of leaf (**B**) and second upper internode (**E**) of the three p2 OE line plants compared with WT plant. Scale bar, 3 cm. Data were shown as mean ± SD (*n* = 3). **G**&**J** An easily broken flag leaf (**G**) and culm (J) of the RGSV-infected rice plant compared with mock rice plant in the mature stage. Scale bar, 3 cm. **H**&**K** The elongation force of leaf (**H**) and second upper internode (**K**) of the RGSV-infected rice plant compared with mock rice plant. Scale bar, 3 cm. Data were shown as mean ± SD (*n* = 3). **I**&**L** The breaking force of leaf (**I**) and second upper internode (**L**) of the RGSV-infected rice plant compared with mock rice plant. Scale bar, 3 cm. Data were shown as mean ± SD (*n* = 3). The significant differences were determined by the Student’s* t*-test (* *p* < 0.05; ** p < 0.01, *** *p* < 0.001)

In addition to reduced plant height, a particularly notable phenotype of the p2 OE plants was the brittleness of their leaves and stems (Fig. 1A and D). To demonstrate this brittleness, we compared the elongation and breaking forces of the leaves and the second internodes between WT and p2 OE transgenic plants. The results showed that the elongation and breaking forces of the p2 OE#1 plant leaves were reduced by 78% and 85%, respectively, compared to those of the WT plants (Fig. 1B and C). Similarly, for the second internodes, the elongation and breaking forces in p2 OE#1 plants were reduced by 80% and 77%, respectively, relative to the WT (Fig. 1E and F). As p2 is a protein encoded by RGSV, we investigated whether RGSV infection results in a similar phenotype. Comparative analysis revealed that RGSV-infected rice leaves and stems also exhibited a noticeable degree of brittleness (Fig. 1G and J). We compared the elongation and breaking forces of the leaves and second internodes between mock- and RGSV-infected rice plants. The elongation and breaking forces of leaves from RGSV-infected plants were reduced by 54% and 62%, respectively, compared with mock plants (Fig. 1H and I). For the second internodes, these values were reduced by 23% and 26%, respectively (Fig. 1K and L). These results indicate that the p2 protein encoded by RGSV can induce dwarfism as well as brittle and abnormal developmental phenotypes in rice.

### Overexpression of RGSV p2 alters cell wall structure by reducing cellulose and increasing lignin accumulation

**Fig. 2:**
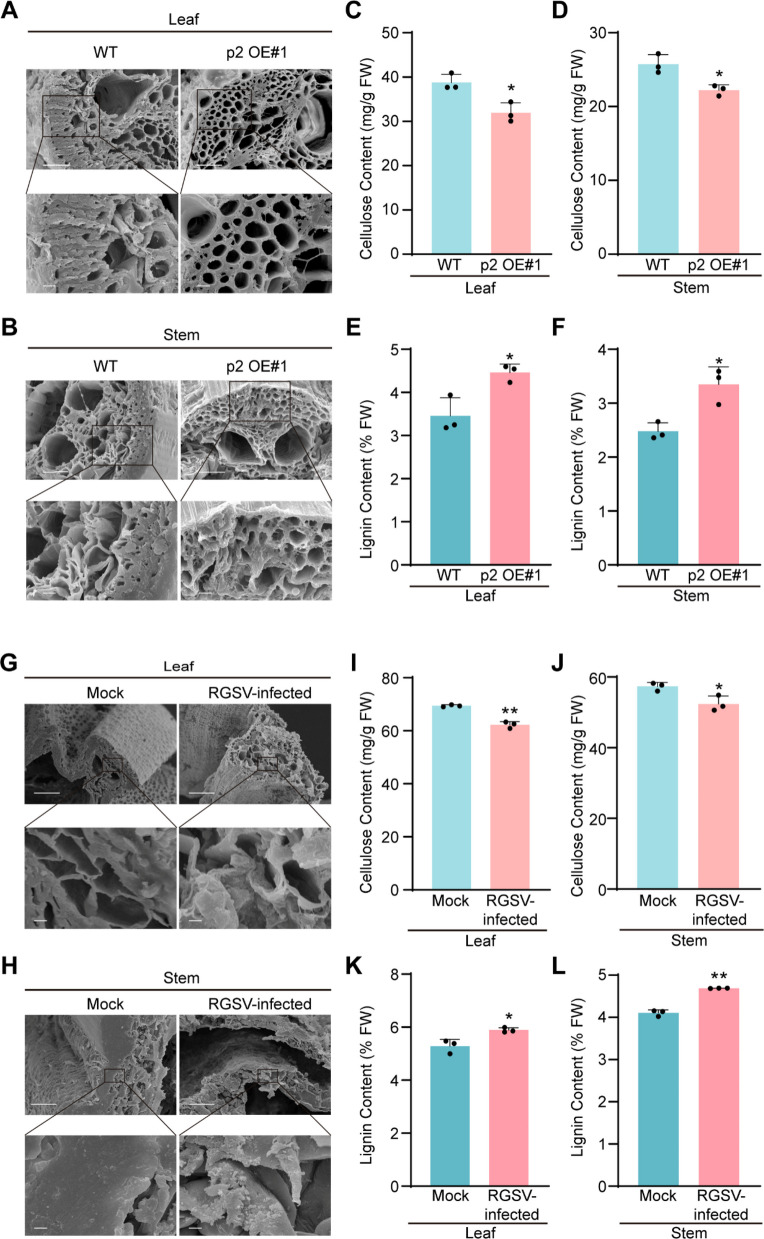
Scanning electron microscopy and cell wall carbohydrate content analyses. **A**&**B** Leaf (**A**) and stem cross-sections (**B**) of the WT and the p2 OE#1 transgenic plants were examined and photographed under a scanning electron microscope. The boxed areas in the upper images are enlarged. Bar = 5 μm (upper panel) and 100 μm (lower panel). Cells within the black boxes are mostly the thickened sclerenchyma cells. **C**&**D** Measurement of cellulose content in the leaves (**C**) and stems (**D**) of the WT and the p2 OE#1 transgenic plants. Data were shown as mean ± SD (*n* = 3). **E**&**F** Measurement of lignin content in the leaves (**E**) and stems (**F**) of the WT and the p2 OE#1 transgenic plants. Data were shown as mean ± SD (*n* = 3). **G**&**H** Leaf (**G**) and stem cross-sections (**H**) of the mock and the RGSV-infected rice plants were examined and photographed under a scanning electron microscope. The boxed areas in the upper images are enlarged. Bar = 5 μm (upper panel) and 100 μm (lower panel). Cells within the black boxes are mostly the thickened sclerenchyma cells. **I**&**J** Measurement of cellulose content in the leaves (**I**) and stems (**J**) of the mock and the RGSV-infected rice plants. Data were shown as mean ± SD (*n* = 3). **K**&**L** Measurement of lignin content in the leaves (**K**) and stems (**L**) of the mock and the RGSV-infected rice plants. Data were shown as mean ± SD (*n* = 3). The significant differences were determined by the Student’s *t*-test (* *p* < 0.05; ** p < 0.01)

To investigate the underlying causes of brittleness and dwarfism in the p2 OE plants, we examined their cell wall structure, given the crucial role of the cell wall in plant height and mechanical strength (Hofte and Voxeur 2017). Scanning electron microscopy of leaf and stem cross‑sections revealed that the sclerenchyma cell walls in p2 OE#1 plants were significantly thinner than those in WT plants (Fig. 2A and B), suggesting a structural weakening. To assess whether the levels of cellulose and lignin, the two main components of the cell wall, were also influenced by p2 expression, we extracted these components from leaf and stem samples of WT and p2 OE#1 plants and quantified them via standard biochemical assays. Cellulose levels were significantly reduced, while lignin levels were markedly increased in the p2 OE#1 plants relative to WT (Fig. 2C–F). Light microscopy analysis of cross-sections from WT and p2 OE#1 plants, stained with calcofluor white, showed a notable reduction in cellulose content in the transgenic lines (Fig. S3A, B, and E). Additionally, scanning electron microscopy was also used to examine cross-sections of RGSV-infected rice tissues, revealing a more relaxed cell wall architecture (Fig. 2G and H). Quantitative analysis revealed that RGSV infection significantly reduced cellulose content and markedly increased lignin accumulation (Fig. 2I–L). Light microscopy of RGSV-infected tissues confirmed a significant reduction in cellulose content compared to WT (Fig. S3C, D, and F). These results indicate that p2 expression significantly alters cell wall architecture, primarily by disrupting cellulose biosynthesis and promoting lignification.

### RGSV p2 partially localizes to the cell wall and accumulates in structural tissues

**Fig. 3:**
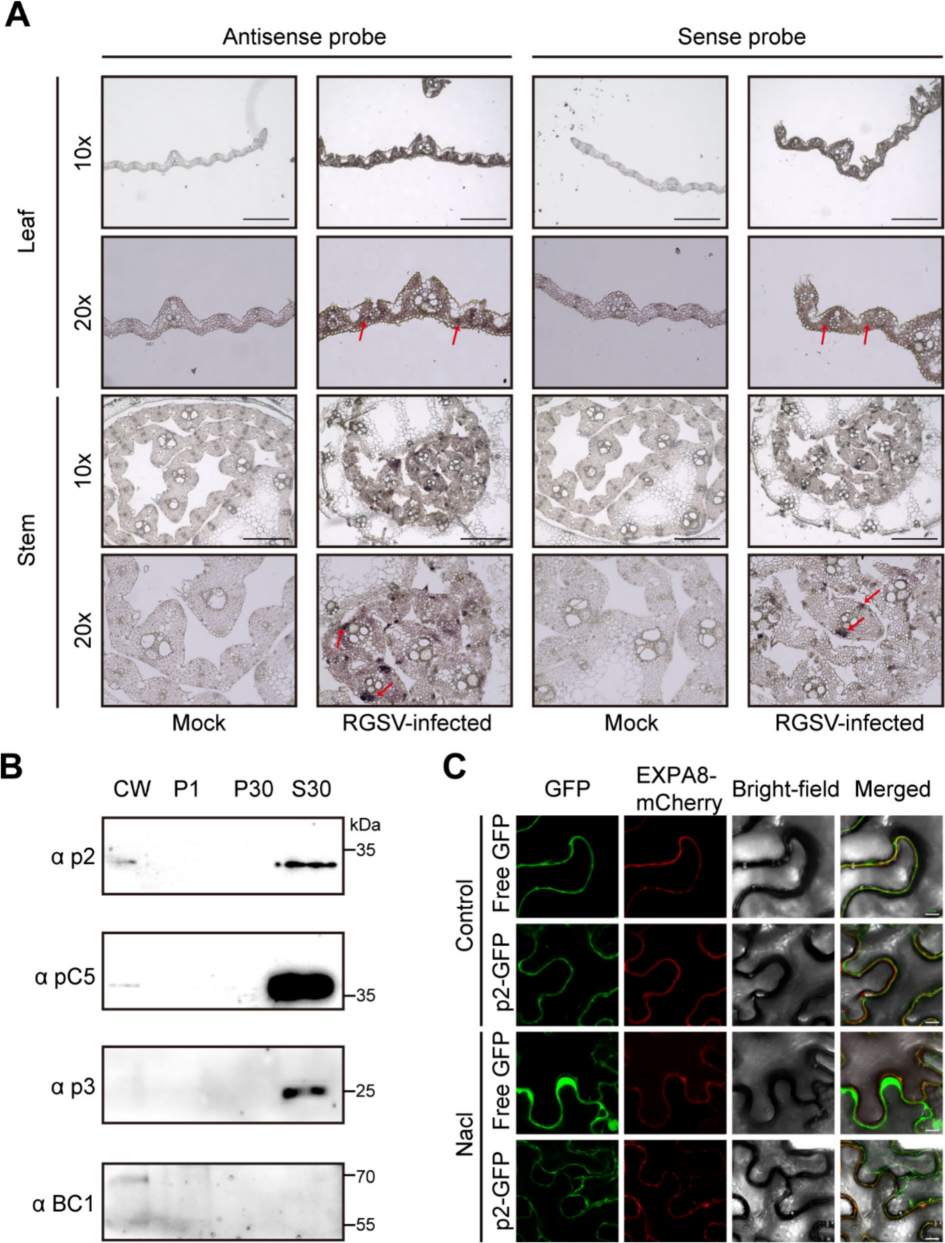
Expression of RGSV p2 in infected rice tissues. **A** Validation of p2 expression. RNA in situ hybridization confirming the expression of vRNA2 in mock and RGSV-infected rice leaf and stem, respectively. Results obtained with sense probe act as negative controls. Red arrows point to areas with high levels of vRNA2 expression. Scale bars, 50 μm. **B** Western blot analysis of leaf homogenates separated into four subcellular fractions (0.5 mg original leaf tissue per lane). Each fraction was probed with anti-p2, anti-CP and anti-p3 antiserum. BC1 served as a cell wall marker. CW, cell wall fraction; P1, organelle-enriched fraction; P30, crude membrane fraction; S30, soluble fraction. **C** Free GFP, p2-GFP, and the cell wall marker EXPA8-mCherry were co-expressed in *Nicotiana benthamiana* epidermal cells. Leaf tissues were imaged before and after treatment with 2.5 M NaCl to induce plasmolysis. Scale bar, 10 µm

To explore the mechanistic basis underlying p2‑induced cell wall brittleness, we examined the subcellular localization of the p2 protein. Transient expression assays in *Nicotiana benthamiana* revealed that p2 localized to both the cytoplasm and nucleus (Fig. S4A), a result further confirmed by nuclear–cytoplasmic fractionation analysis (Fig. S4B). Given that p2 expression disrupts cell wall structure and composition in rice, we next investigated its spatial distribution within infected tissues. RNA in situ hybridization on RGSV‑infected rice revealed widespread expression of p2 transcripts, with strong signals detected in fundamental and mechanical tissues (Fig. 3A), suggesting a possible association with cell wall‑related structures. To further explore this possibility, we isolated four subcellular fractions from RGSV‑infected rice leaves: the cell wall fraction (CW), the organelle‑enriched fraction (P1), the crude membrane fraction (P30), and the soluble cytoplasmic fraction (S30). Immunoblot analysis showed that although the majority of p2 protein was enriched in the S30 fraction, a detectable amount was also present in the CW fraction. Importantly, the CW fraction was validated using the cell wall–associated protein BC1 (Liu et al. 2013), which was strongly enriched in the CW fraction but absent from the S30 and P1 fractions. In contrast, the control viral protein P3 was detected only in the S30 fraction and was not present in the CW fraction (Fig. 3B).

To further assess whether p2 can physically associate with the cell wall, we performed plasmolysis assays in *Nicotiana benthamiana* epidermal cells by co-expressing free GFP, p2-GFP, and the cell wall marker EXPA8–mCherry (Wang et al. 2014), followed by treatment with 2.5 M NaCl. After plasmolysis, free GFP fully retracted into the protoplast, whereas p2-GFP retained partial co-localization with EXPA8–mCherry at the cell wall (Fig. 3C). These observations are consistent with the biochemical fractionation results and support the presence of a minor cell wall–associated pool of p2.

Collectively, these findings indicate that although p2 predominantly localizes to the cytoplasm and nucleus, a small but reproducible fraction associates with the cell wall and accumulates in structural tissues, which may contribute to the observed alterations in cell wall integrity.

### Transcriptome analysis reveals candidate pathways underlying brittle phenotype and enhanced immunity

**Fig. 4:**
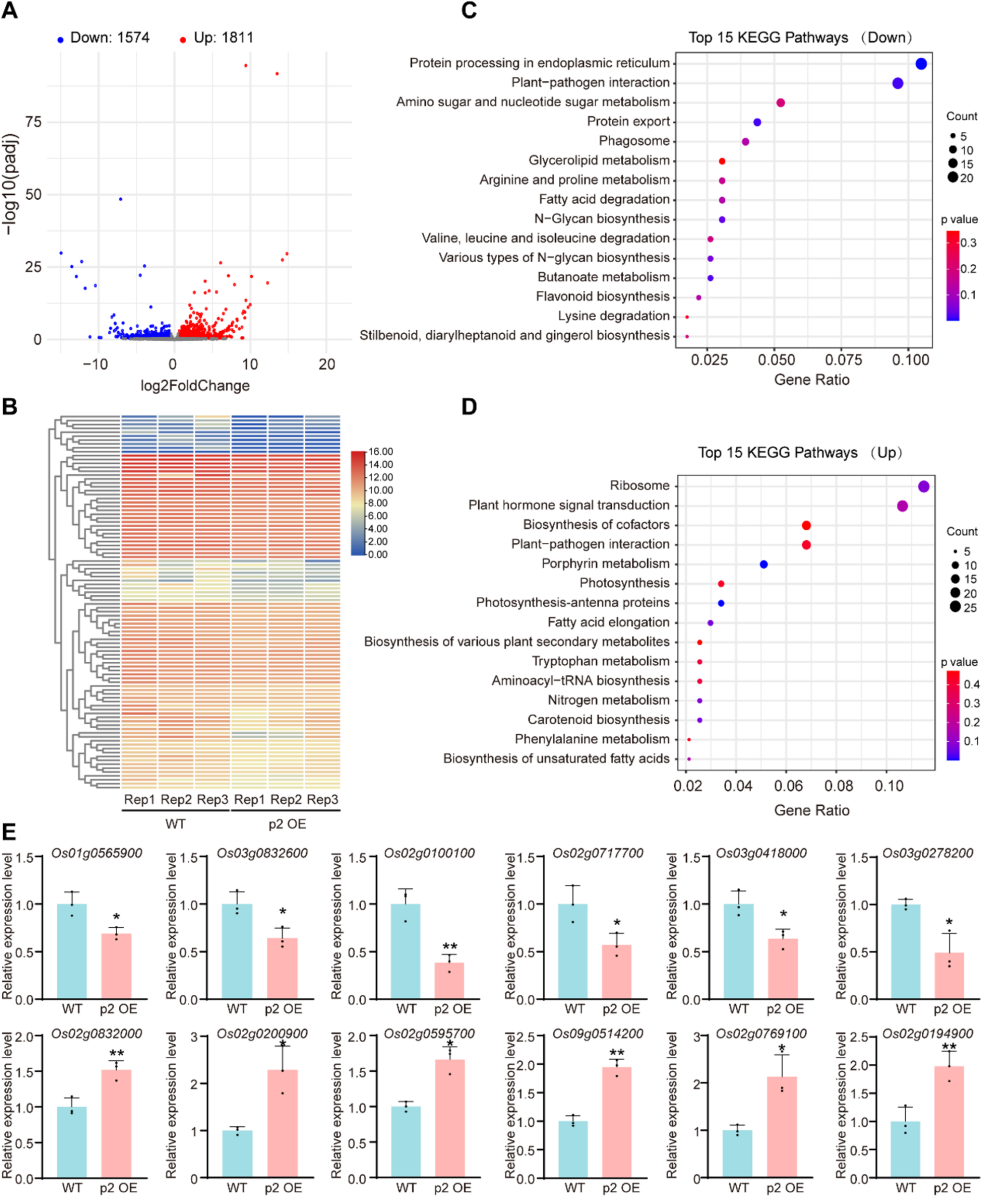
Transcriptomic profiling of WT and p2 OE rice plants. **A** Volcano plot showing differentially expressed genes (DEGs) between WT and p2 OE plants. Red and blue dots represent significantly upregulated and downregulated genes, respectively (|log₂ fold change|≥ 1, FDR < 0.05). **B** Heatmap displaying hierarchical clustering of gene expression profiles across three biological replicates of WT and p2 OE plants. **C** KEGG pathway enrichment analysis of the top 15 downregulated gene categories. **D** KEGG pathway enrichment analysis of the top 15 upregulated gene categories. **E** Quantitative real-time PCR validation of selected upregulated and downregulated genes identified in the RNA-seq dataset. Gene expression levels were normalized to *OsEF-1α* and shown as means ± SD of three biological replicates. The significant differences were determined by the Student’s *t*-test (* *p* < 0.05; ** *p* < 0.01)

To gain deeper insight into the molecular basis of the brittle and stunted phenotypes observed in p2 OE plants, we performed transcriptome profiling using RNA-seq analysis samples from 42-day-old WT and p2 OE plants. Principal component analysis confirmed clear transcriptomic separation between WT and p2 OE samples, each with three biological replicates (Table S1). A volcano plot showed a total of 1574 downregulated and 1811 upregulated genes (Fig. 4A), and a heatmap displayed distinct gene expression patterns between WT and p2 OE transgenic lines (Fig. 4B). KEGG pathway enrichment analysis revealed that downregulated genes were significantly enriched in "protein processing in endoplasmic reticulum", "plant-pathogen interactions", and "amino sugar and nucleotide sugar metabolism" (Fig. 4C). Interestingly, these pathways are closely related to cell wall biosynthesis and plant immunity. In contrast, the top three enriched pathways among upregulated genes were "ribosome", "plant hormone signal transduction", and "biosynthesis of cofactors", indicating potential compensatory responses or altered hormonal signaling (Fig. 4D). To validate the RNA-seq results, we selected representative differentially expressed genes from both upregulated and downregulated groups and confirmed their expression patterns using quantitative real-time PCR (RT-qPCR) (Fig. 4E). Among the downregulated genes, NAD dependent epimerase/dehydratase family domain containing protein (*Os03g0278200*), GHMP kinases ATP-binding proteins (*Os03g0832600*), and protein disulfide isomerase OsPDIL1-4 (*Os02g0100100*) have been previously implicated in sugar metabolism, protein folding in the endoplasmic reticulum, and acetylation of structural macromolecules, all essential processes for cell wall assembly and remodeling (Bar-Peled and O'Neill 2011; Yin et al. 2011; Onda and Kobori 2014). In contrast, upregulated genes included OsEBF2 (*Os02g0200900*), a negative regulator of ethylene signaling, positively regulate rice resistance to BPH (Ma et al. 2023). These findings suggest that p2 expression affects both structural and defense-related pathways at the transcriptional level, which may underlie the observed mechanical weakness and altered pathogen/herbivore responses.

Given that “plant-pathogen interaction” and hormone-related pathways were affected in p2 OE plants, we further hypothesized that p2 expression might alter host susceptibility not only to viruses but also to phloem-feeding insects such as BPHs. To test this, we conducted a comprehensive series of assays comparing the insect performance on WT and p2 OE rice plants. In a BPH host preference assay, significantly fewer insects were observed on p2 OE plants beginning at 48 h post-inoculation (hpi) (Fig. S5A and B). Additionally, BPH oviposition was markedly reduced on p2 OE lines compared to WT, as revealed by both egg photography and oviposition counts (Fig. S5C and D). Honeydew excretion, an indicator of insect feeding activity, was also significantly lower on p2 OE plants (Fig. S5E and F). Importantly, survival assays revealed that p2 OE plants exhibited greater resistance to BPH inoculation, with higher plant survival at 8 days post-inoculation (dpi) compared to both WT and the BPH-susceptible variety TN1 (Fig. S5G). These results demonstrate that p2 OE plants exhibit enhanced resistance to BPH, further supporting the hypothesis that p2-induced cell wall remodeling not only affects structural integrity but also modulates rice interaction with insect herbivores.

### Overexpression of RGSV p2 promotes broad‑spectrum viral susceptibility in rice

**Fig. 5:**
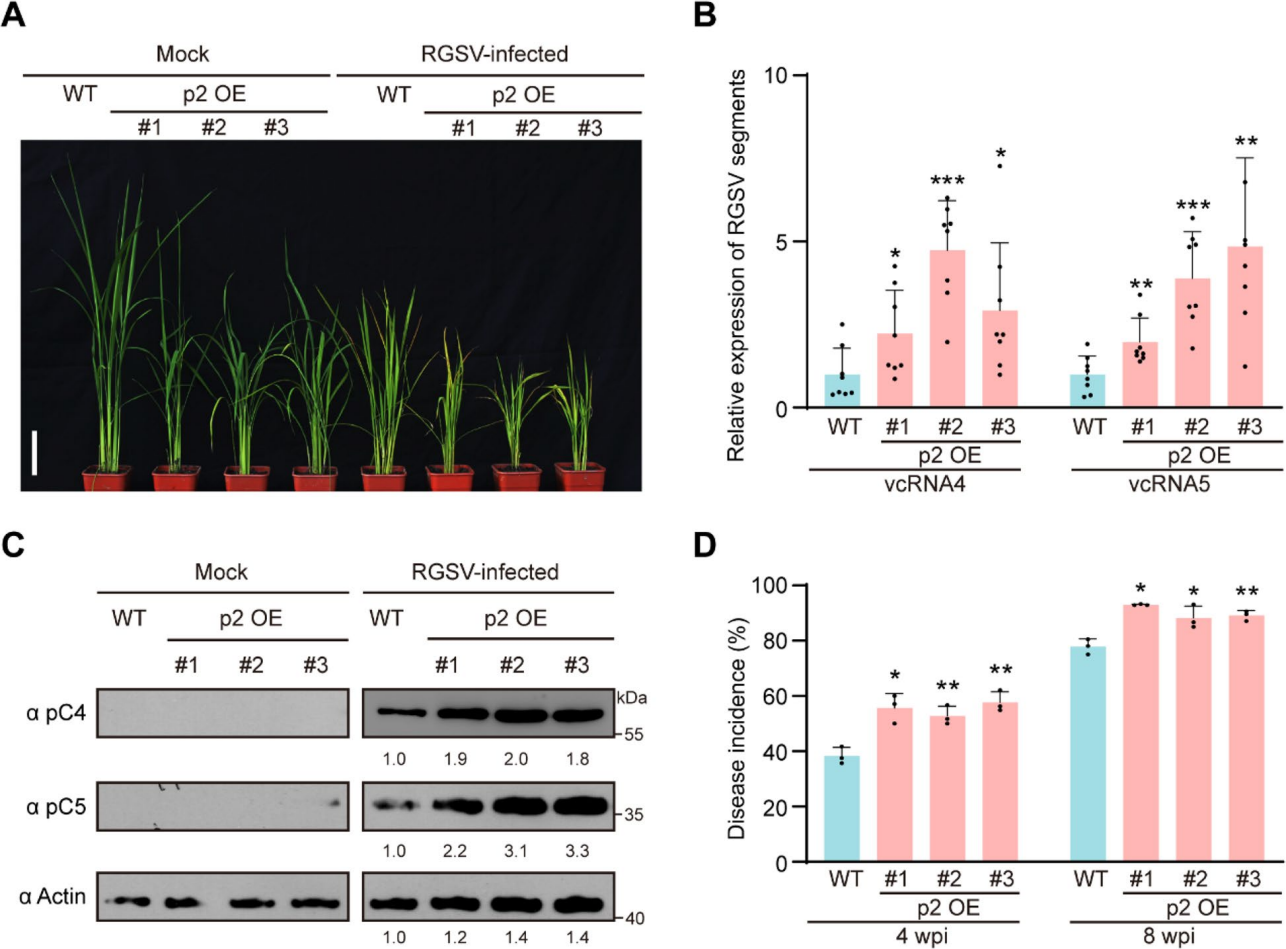
The p2 OE transgenic lines are more susceptible to RGSV infection. **A** Photographs of mock or RGSV-infected WT and p2 OE transgenic rice lines. Scale bar, 10 cm. Photos were taken at 4 weeks post-inoculation (wpi). **B** Quantitative real-time PCR analysis of RGSV vcRNA4 and vcRNA5 RNA accumulation in the indicated plants. Data were presented as mean ± SD (*n* = 8). **C** Immunoblot analysis of RGSV pC4 and pC5 levels in the indicated plants. Actin was used as an internal control. **D** Disease incidence of rice plants infected with RGSV at 4 wpi and 8wpi. Data were shown as mean ± SD (*n* = 3). The significant differences were determined by the Student’s *t*-test (* *p* < 0.05; ** *p* < 0.01, *** *p* < 0.001)

Plant cell walls serve not only as structural frameworks but also as critical barriers against pathogens (Kesten et al. 2017). To investigate whether RGSV infection is influenced by alterations in cell wall structure and composition in p2 OE transgenic plants, we inoculated 2-week-old WT seedlings and p2 OE transgenic lines with RGSV-carrying brown planthoppers, subsequently monitoring disease symptom development. By 4 weeks post virus inoculation (wpi), all plants of the three p2 OE transgenic lines exhibited symptoms of viral infection and were noticeably more stunted than the WT control plants (Fig. 5A), suggesting that overexpression of the p2 protein in rice enhances susceptibility to RGSV. RT-qPCR and Western blot assays revealed that higher levels of RGSV genomic RNAs and proteins accumulated in the RGSV-inoculated p2 OE plants compared to the inoculated WT plants (Fig. 5B and C). Moreover, infection rates across multiple time points post‑inoculation were significantly higher in p2 OE lines (Fig. 5D). To assess whether p2 overexpression confers broad-spectrum susceptibility to viral infections, we inoculated WT and p2 OE transgenic plants with either RDV or SRBSDV, both of which are double-stranded RNA viruses. Compared to WT plants, p2 OE transgenic plants exhibited more severe symptoms and accumulated higher levels of viral RNAs and proteins following RDV or SRBSDV inoculation (Fig. S6). Together, these data demonstrate that RGSV p2 overexpression promotes broad‑spectrum viral susceptibility in rice, linking cell wall disruption to enhanced pathogen invasion.

### Cell wall-deficient rice mutants exhibit enhanced virus susceptibility

**Fig. 6:**
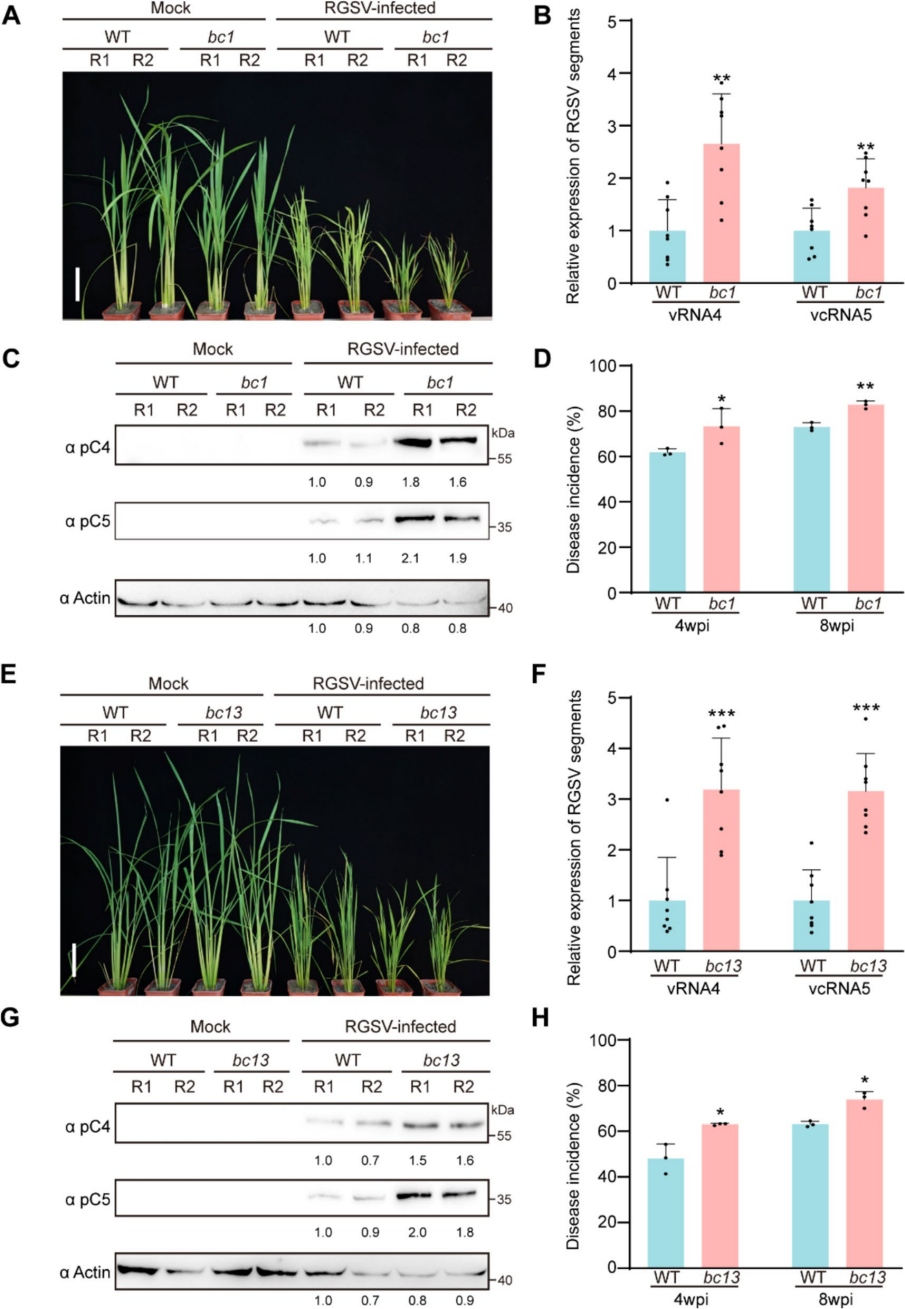
The brittle mutant rice plants are also more susceptible to RGSV infection. **A**&**E** Photographs of mock or RGSV-infected WT and *bc1* (**A**) or *bc13* (**E**) rice lines. Scale bar, 10 cm. Photos were taken at 4 wpi. **B**&**F** Quantitative real-time PCR analysis of RGSV vcRNA4 and vcRNA5 RNA accumulation in the indicated plants. Data were presented as mean ± SD (*n* = 8). **C**&**G** Immunoblot analysis of RGSV pC4 and pC5 levels in the indicated plants. Actin was used as an internal control. **D**&**H** Disease incidence of rice plants infected with RGSV at 4 wpi and 8wpi. Data were shown as mean ± SD (*n* = 3). The significant differences were determined by the Student’s *t*-test (* *p* < 0.05; ** *p* < 0.01, *** *p* < 0.001)

In the studies above, we have demonstrated that the RGSV p2 protein alters cell wall composition in a manner that facilitates viral infection. To further investigate the relationship between cell wall morphology and viral susceptibility in rice, we analyzed two brittle rice mutants, *bc1* and *bc13*. The *BC1* gene encodes a COBRA-like protein and is expressed in sclerenchyma cells and vascular bundles during rice development. Mutations in this gene result in reduced cell wall thickness and cellulose content, and increased lignin accumulation, leading to brittle stems and leaves (Li et al. 2003; Liu et al. 2013). In contrast, the *bc13* mutant harbors a G551A missense mutation in *OsCESA9* gene (cellulose synthase 9). This mutation causes a glutamic acid to lysine substitution within an acidic amino acid-rich region following the N-terminal zinc finger domain, resulting in mechanical strength approximately one-third that of the WT, thinner cell walls, and a 22% reduction in cellulose content compared to WT plants (Song et al. 2013). We inoculated these mutant rice plants with RGSV using viruliferous brown planthoppers. By 4 wpi, the RGSV-inoculated *bc1* and *bc13* mutants exhibited more pronounced stunting than RGSV-inoculated WT plants (Fig. 6A and E). Results of RT-qPCR and Western blot assays showed that the levels of RGSV RNAs and proteins were significantly higher in these mutants than in the infected WT plants (Fig. 6B, C, F and G). Furthermore, the RGSV infection rates of these mutant lines were higher than those of the WT plants at multiple time points post-inoculation (Fig. 6D and H). Because modifications in cell wall components may also influence the interaction between rice and its insect vector, we additionally evaluated the performance of BPHs on *bc1* and *bc13* (Fig. S7). Although the detailed trends differed slightly between the two mutants, both exhibited altered insect responses compared with WT plants, indicating that cell wall defects can influence insect behavior to some extent. These findings collectively support the hypothesis that cell wall-induced brittleness contributes to broad-spectrum viral susceptibility.

## Discussion

### RGSV p2 as a viral effector that weakens host structural defenses

Cell wall integrity is not only critical for disease resistance but also represents an important agronomic trait in plants (Bacete et al. 2018; Houston et al., 2016; Kesten et al. 2017). Culm strength is a major determinant of lodging resistance, which directly affects grain yield, milling quality, and cultivar adaptability (Ana et al. 2022, Mengistie and McDonald 2023). Mutations causing brittle culms often lead to severe lodging, reducing harvest efficiency and compromising production (Li et al. 2003; Tanaka et al. 2003; Yan et al. 2007; Zhang et al. 2009, 2010; Zhou et al. 2009; Hirano et al. 2010). This study reveals that the RGSV-encoded p2 protein plays a pivotal role in manipulating host cell wall architecture. Transgenic overexpression of p2 in rice results in a brittle phenotype and stunted growth, closely mirroring symptoms observed during RGSV infection (Fig. 1). Microscopic imaging and biochemical quantification demonstrated that p2 reduces cellulose content while increasing lignin accumulation, suggesting interference with normal wall polymer biosynthesis (Fig. 2). These effects were further supported by the detection of p2 in purified cell wall fractions and its partial localization to the cell periphery, implying physical association with wall structures (Fig. 3). Viral suppression of host physical barriers has been observed in other systems, but this is important evidence of a *tenuivirus* protein directly contributing to wall remodeling. These findings position p2 as a novel structural effector that facilitates viral adaptation by weakening host mechanical resilience.

Interestingly, alterations in cell wall composition may have additional applications beyond crop protection. Previous studies have shown that brittle culm mutants, due to their modified cell wall composition, exhibit increased palatability and digestibility, making them promising candidates for livestock feed (Su et al. 2012; Halpin 2019; Yi et al. 2022). Furthermore, such modifications may enhance the efficiency of biomass conversion into bioethanol, highlighting the potential value of brittle rice lines as feedstock for renewable energy production (Wu et al. 2013; Ningthoujam et al. 2023; Li et al. 2024). Our study shows that p2-overexpressing plants, as well as *bc1* and *bc13* mutants, exhibit pronounced brittleness and reduced viral resistance (Figs. 5 and 6), underscoring the dual roles of cell wall structure in agronomic performance and pathogen defense. The dual implications of our findings—in disease susceptibility and biomass utility—suggest that careful manipulation of cell wall traits could serve both agronomic and industrial goals.

### Disruption of wall integrity and its signaling consequences

Our transcriptomic analyses reveal that p2 expression reprograms gene networks involved in wall biosynthesis, ER protein processing, hormone signaling, and pathogen perception (Fig. 4). These changes echo the hallmarks of disrupted cell wall integrity (CWI), a phenomenon increasingly recognized as a trigger for compensatory stress responses. In Arabidopsis, damage to the primary wall can activate wall-associated kinases (WAKs), receptor-like kinases (RLKs), and MAPK cascades to reinforce defense (Decreux and Messiaen 2005; Kohorn et al. 2009; Brutus et al. 2010). It is plausible that the altered expression of defense-related genes in p2 OE lines is a downstream consequence of wall stress perception. Whether p2 directly modulates CWI signaling components or indirectly induces their activation remains unknown. Furthermore, the upregulation of ribosomal genes and hormone pathways (e.g., jasmonic acid, auxin, ethylene) in p2 OE plants could reflect broader adjustments to disrupted growth equilibrium. These observations suggest that the function of p2 may extend beyond structural degradation to include the manipulation of signaling hubs that coordinate wall maintenance, growth, and immunity. Future studies employing phosphoproteomics and interact omics will be crucial to identify whether p2 interfaces with known cell wall surveillance machinery or induces novel signaling routes.

### Ecological and evolutionary implications of structural remodeling

An unexpected outcome of p2-mediated wall remodeling is the enhanced resistance to the BPH, the vector of RGSV. This observation reveals a fascinating trade-off: the same wall compromise that facilitates viral entry appears to hinder insect colonization. We propose that changes in wall stiffness, lignin deposition, or sugar metabolism alter the physical and nutritional landscape of host tissues, thereby impairing phloem access or feeding efficiency. This raises broader questions about how viral adaptations may generate ecological ripple effects beyond their primary host targets. While RGSV may benefit from host wall weakening in the short term, increased resistance to its vector could potentially reduce transmission opportunities, representing an evolutionary constraint. Such feedback loops may shape virus evolution in ways not solely dictated by host compatibility (Doumayrou et al. 2013; Long et al. 2018; Zhu et al. 2021). Notably, similar antagonistic effects have been reported in virus-induced dwarf or lesion mimic mutants, where enhanced basal defenses come at a cost to other interactions (Zeng et al. 2004; Huot et al. 2014). Thus, p2 exemplifies how structural remodeling can mediate both pathogenic success and ecological consequence, reinforcing the importance of considering host traits as nodes in multi-partner networks.

## Conclusions

**Fig. 7:**
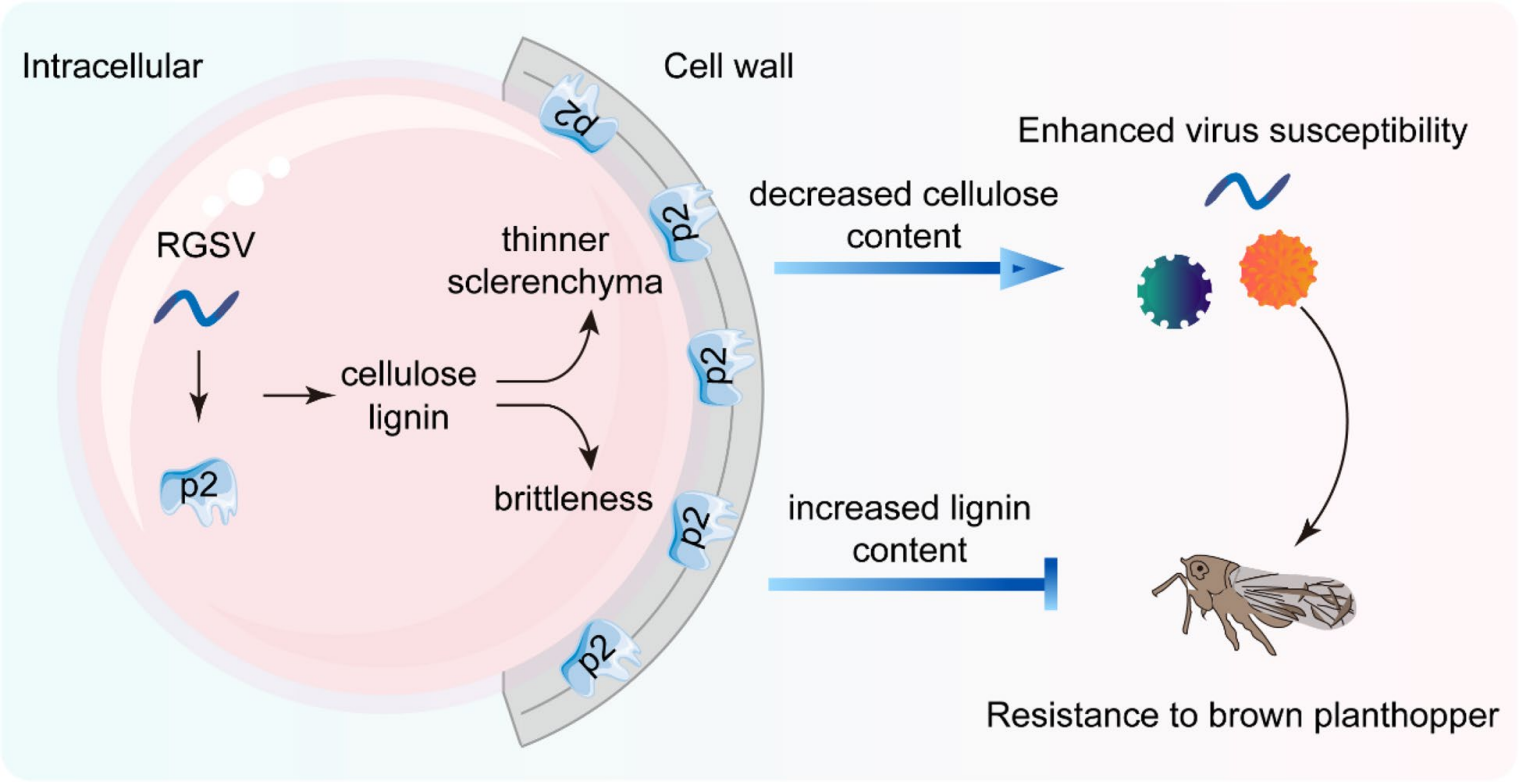
A proposed working model of RGSV p2-mediated cell wall remodeling and host susceptibility modulation. The schematic illustrates the proposed function of RGSV-encoded p2 protein in rice. Upon expression, p2 partially localizes to the cell wall and interferes with cellulose biosynthesis while enhancing lignin accumulation, leading to a brittle and weakened cell wall structure. These changes compromise mechanical strength and increase plant susceptibility to virus infection. In contrast, the altered cell wall properties appear to reduce phloem sap accessibility, thereby limiting brown planthopper feeding and oviposition. Arrows indicate positive effects; blunt-ended lines indicate negative effects

Based on our results, we propose a possible working model of p2 function in rice-virus interaction (Fig. 7). In summary, our findings identify RGSV p2 as a unique viral effector that targets cell wall metabolism to reshape rice interactions with both viruses and herbivores. By dissecting the structural, transcriptomic, and ecological impacts of p2 expression, this study offers new mechanistic insights into virus-driven manipulation of host architecture. It also positions cell wall integrity as a dual-purpose trait—governing both susceptibility and ecological fitness—with implications for breeding crops resilient to diverse biotic stresses.

## Materials and methods

### Plasmid construction and rice transformation

Full length open reading frame of RGSV vRNA2 was amplified through RT-PCR and the resulting fragment was cloned into the *pCAMBIA2300:Actin:OCS* vector to produce *pCAMBIA2300:Actin:vRNA2*. The final construct was transformed into rice cv. Nipponbare using an Agrobacterium-mediated transformation method by the BioRun Co., Ltd., Wuhan, China. Primers used in RT-PCR analyses are listed in the Table S2.

### Plant growth and virus inoculation

Plants were grown and inoculated with viruses as previously described (Wu et al. 2025). Specifically, rice seedlings were grown inside a greenhouse maintained at 28 °C–30 °C and a 60 ± 5% relative humidity. Rice seedlings at the third-leaf stage were inoculated with 2 to 3 RGSV viruliferous brown planthoppers per seedling for 3 days. The brown planthoppers were removed and the rice seedlings were again grown inside the greenhouse under the same growth conditions mentioned above. At 4 weeks post virus inoculation (wpi), the inoculated plants were photographed and then harvested for further analyses. A minimum of 30 rice seedlings were used for each treatment. For each assayed rice line, the number of plants showing virus symptoms was recorded weekly and the disease incidence were calculated and analyzed.

### Brown planthopper inoculation and resistance assay

BPH performance on rice plants was evaluated using host choice, BPH oviposition assay, and honeydew excretion as previously described (Shi et al. 2021) with minor modifications. For analysis of host choice, seeds of each line were sown in a plastic cup (10-cm-diameter). At the 3-leaf stage, the cup was covered with nylon mesh and 5 second- to third-instar BPH nymphs per plant were released into the cup. The number of BPHs that settled on each plant was recorded at 3, 6, 12, 24, 48, 72, 96, and 120 h post-inoculation (hpi). For the oviposition assay, approximately fourth-instar nymphs were allowed to develop into a mating pair. One male and one female BPH were released onto an individual rice plant at the seedling stage and enclosed using a plastic cup as a cage. After 10 days of inoculation, plants were dissected and the total number of eggs laid inside the culms was counted manually. For the honeydew excretion assay, plants were placed individually in plastic cup chamber, each with an opening at the base and the top. A single rice seedling was passed through the chamber, and the top hole was sealed with a cotton plug around the plant base. 5 third-instar BPH nymphs were introduced into each chamber. A piece of Whatman No. 1 filter paper was placed at the base to absorb honeydew droplets. After 48 h of feeding, the filter papers were removed and stained using ninhydrin solution. The honeydew-stained area was imaged and quantified using ImageJ software.

Resistance levels of rice populations to BPH were evaluated at the seedling stage, eight third-instar BPH nymphs were released on each seedling at the 3-leaf stage. Plants were kept in insect-proof cages and photographed at 0 and 8 days post-inoculation (dpi). The susceptible variety TN1 was used as a control. Assay was performed with at least three biological replicates, and each replicate consisted of 24 plants per line.

### Measurement of mechanical strength

At the heading stage, the flag leaf and the second internode of the WT plants and the p2 OE transgenic rice plants were harvested and cut into equal length segments. The breaking force of each harvested segments was measured using a micro force/length testing Instron 5848 MicroTester device as previously described (Wang et al. 2012).

### Light microscopy and scanning electron microscopy

At the heading stage, the fresh flag leaf and the second internode were harvested from the WT and the p2 OE transgenic rice plants, cut into small segments, and then fixed in a fixative solution containing 75% ethanol, 5% acetic acid, 5% glycerol and 5% formaldehyde for at least 1 day. The fixed leaf sheath samples were dehydrated in a graded ethanol solution, incubated overnight in a xylene solution, rinsed several times with ethanol, and then observed under a light microscope. For scanning electron microscopy, the fixed tissue samples were dried to a critical point, sputter-coated with gold, and then observed under a S-2380N scanning electron microscope (Hitachi, Tokyo, Japan) as previously described (Wang et al. 2016).

### Measurement of cellulose and lignin contents

Leaf and stem samples were collected from WT plants and p2 OE lines at the heading stage, after which their cellulose contents were measured using Anthrone-Sulfuric Acid method as previously described and modified (Updegraff 1969). Accurately weigh 0.15 g of sample, put it into a 25 ml volumetric flask, which was then put into an ice bath, add 10–20 ml of cold 60% H_2_SO_4_, digested under ice bath for half an hour, then diluted to scale with 60% H_2_SO_4_, shaken well, and filtered with a glass crucible. Suck 5.0 ml of the above filtrate, put it into a new 25 ml volumetric flask in ice bath, add distilled water to release it to the scale, shake well. Take 2 ml of the upper solution, add 0.5 ml of 2% anthrone reagent, add 5 ml of concentrated H_2_SO_4_ along the wall of the tube, and cover the plug. The extinction value of the sample at 620 nm is measured.

Additionally, the leaf and stem lignin contents were measured according to a published method and modified (Dence 1992). Weigh 0.1–0.25 g of plant materials into a centrifuge tube, add 10 ml of 1% acetic acid solution, shake well and centrifuge; wash the precipitate once with 5 ml of 1% acetic acid, then add 3–4 ml of ethanol and ether to mix (volume ratio 1:1), soak for 3 min, discard the supernatant and wash for three times. Evaporate the precipitate in the centrifuge tube in the boiling water bath, then add 3 ml of 72% sulfuric acid to the precipitate, mix it with a glass rod, leave it at room temperature for 16 h to dissolve all cellulose, then add 10 ml distilled water to the tube, mix it with a glass rod, put it in the boiling water bath for 5 min, add 5 ml distilled water and 0.5 ml 10% barium chloride solution after cooling, shake well and centrifuge. The precipitate was washed twice with distilled water, and then 10 ml sulfuric acid and 0.1 mol/L potassium dichromate solution were added to the washed lignin precipitate. The tube was put into boiling water bath for 15 min and stirred at any time. After cooling, transfer all substances in the test tube to the beaker for titration, and wash the residual part with 15–20 ml distilled water. Then add 5 ml of 20% KI solution and 1 ml of 0.5% starch solution into the beaker, and titrate with 0.2 mol/L sodium thiosulfate.

### Cellulose staining using Calcofluor White

Leaf and stem tissues were hand-sectioned and fixed in FAA solution (50% ethanol, 5% acetic acid, and 3.7% formaldehyde) for at least 12 h at room temperature. After rinsing with distilled water, the samples were incubated in 0.1% Calcofluor White solution (Sigma-Aldrich) for 15 min in the dark. Stained sections were rinsed twice with distilled water and mounted in water on glass slides. Cellulose-associated fluorescence was visualized using a fluorescence microscope with a DAPI filter set (excitation ~ 365 nm; emission ~ 435–480 nm). Fluorescence intensity was quantified using ImageJ software.

### RNA in situ hybridization

In situ hybridization was performed as described previously (Zhang et al. 2021). Digoxigenin-labeled hybrid probes were transcribed in vitro from the coding sequence encoding the RGSV p2 protein with gene-specific primers (Table S2). Leaf and tem cross-sections were used for hybridization assays. Slides were photographed under a microscope (Nikon SMZ25, Nikon Instruments, Tokyo, Japan).

### Plasmolysis assay

*Agrobacterium* carrying p2-GFP, free GFP, or EXPA8–mCherry were prepared in infiltration buffer (OD₆₀₀ = 0.5), mixed at a 1:1 ratio, and infiltrated into *Nicotiana benthamiana* leaves. After 36 h, leaf discs were mounted in ddH₂O (control) or incubated in 2.5 M NaCl to induce plasmolysis, followed by confocal imaging.

### RNA-seq analysis

Total RNA was extracted from 42-day-old WT or p2 OE transgenic rice plants using a RNeasy Plant Mini kit (Qiagen, Hilden, Germany). The subsequent RNA-seq analyses were performed by the Wuhan igenebook Co., Ltd. (Wuhan, China). Libraries with adapter-ligated fragments were paired-end sequenced to generate reads with 150 nucleotides. The FastQC software was used to assess the quality of the raw reads. After removing the adapters and low-quality reads, the clean reads were used to search the MSU7.0 rice reference genome using the TopHat software and then analyzed with the Cufflinks as previously described (Trapnell et al. 2012). The Poisson fragment dispersion model was used for the statistical analyses (FDR < 0.05). Differentially expressed genes (DGEs) were identified based on a greater than 1.5-fold difference in the reads per kilobase per million reads (RPKM). Three biological replicates were used for this analysis and their repeatability and correlations were evaluated according to the Pearson’s correlation coefficient (Nora et al. 2012).

### RT-PCR and qRT-PCR

Total RNA (2 µg) was used in reverse transcriptions with a SuperScript III Reverse Transcriptase (Invitrogen, Carlsbad, CA, USA). For qRT-PCR, each 20 μL reaction contained 4 µL 20-fold diluted cDNA, 0.5 µM each primer and 10 µL SYBR Green PCR Master Mix (Lablead, China). The gene expression levels were normalized against the expression level of *OsEF-1α*. Primers used in these assays are listed in the Table S2.

### Statistics analysis

Statistical analyses were conducted using the Student’s *t*-test (* *p* < 0.05; ** *p* < 0.01, *** *p* < 0.001, **** *p* < 0.0001). All data are presented as means ± SD of at least three biological replicates as indicated.

## Supplementary Information

Supplementary Material 1: Figure S1. Phenotype of WT and p2 OE rice plants. (A) Morphology of WT and p2 OE transgenic rice lines. Scale bar, 15 cm. (B) Tillering number comparison. Data were presented as mean ± SD (*n* = 10). (C) Productive ear number comparison. Data were presented as mean ± SD (*n* = 10). (D) 1000-grain weight comparison. Data were presented as mean ± SD (*n* = 10). (E) Statistical analysis of plant height in WT and p2 OE transgenic rice lines. Data were presented as mean ± SD (*n* = 10). (F) Grain shape comparison. Ten well-filled grains are presented in a column. Scale bar, 1 cm. (G) Comparison of grain length. Data were presented as mean ± SD (*n* = 100). (H) Comparison of grain width. Data were presented as mean ± SD (*n* = 100). The significant differences were determined by the Student’s* t*-test (*** *p* < 0.001). Figure S2. The length of cells and internodes of the WT and the p2 OE transgenic plants. (A) An image showing stems of the WT and the p2 OE#1 plants. Arrowheads indicate the stem nodes. I–IV, the first to the fourth internodes. The image was taken at 42-day post-planting. Scale bar, 10 cm. (B) The length of individual internodes relative to the total culm length. (C) Microscopic images showing the length of cells in the sheath of the WT and the three p2 OE transgenic lines. Scale bar, 300 µm. (D) Statistical analysis of cell length. Data were presented as mean ± SD (*n* = 100). The significant differences were determined by the Student’s *t*-test (** *p* < 0.01, *** *p* < 0.001). Figure S3. Staining of cellulose in leaf and stem sections. (A&C) Images showing cellulose contents in leaf sections from the WT and the p2 OE#1 plants (A) or mock and RGSV-infected plants (C). Cellulose in the leaf sections was stained with Calcofluor white. Scale bar, 50 μm. (B&D) Images showing cellulose contents in stem sections from the WT and the p2 OE#1 plants (B) or mock and RGSV-infected plants (D). Cellulose in the leaf sections was stained with Calcofluor white (E) Statistical analysis of cellulose staining fluorescence intensity in leaves and stems of WT and p2 OE#1 plants. Data were shown as mean ± SD (*n* = 3). (F) Statistical analysis of cellulose staining fluorescence intensity in leaves and stems of mock and RGSV-infected plants. Data were shown as mean ± SD (*n* = 3). The significant differences were determined by the Student’s *t*-test (* *p* < 0.05; ** *p* < 0.01). Figure S4. Subcellular localization of p2. (A) Subcellular localization of RGSV p2 in *Nicotiana benthamiana* leaf cells. Scale bar, 10 µm. (B) RGSV p2 was detected in the cytoplasmic protein fraction through Western blot assay. Actin and H3 in the fractions were also detected and used as the markers for cytoplasm and nuclear, respectively. Figure S5. Performance of BPH on WT and p2 OE rice plants. (A) Photographs showing the distribution of BPH individuals on WT and p2 OE rice plants at 72 h post-inoculation (hpi). (B) Time-course analysis of the number of BPH individuals settled on WT and p2 OE plants at 6, 12, 24, 48, 72, 96, and 120 hpi. Data were shown as mean ± SD (*n* = 3). (C) Photographs of BPH oviposition on WT and p2 OE plants stained to visualize egg deposition. (D) Quantification of the number of eggs laid by BPH on WT and p2 OE plants. Data were shown as mean ± SD (*n* = 6). (E) Representative images of honeydew excretion spots produced by BPH feeding on WT and p2 OE plants. (F) Quantification of honeydew area on WT and p2 OE plants as a proxy for insect feeding activity. Data were shown as mean ± SD (*n* = 15). (G) Photographs of WT, p2 OE, and susceptible control TN1 rice plants before (0 day) and after (8 days) BPH inoculation. Scale bar, 5 cm. The significant differences were determined by the Student’s *t*-test (* *p* < 0.05; ** *p* < 0.01, *** *p* < 0.001, **** *p* < 0.0001). Figure S6. The p2 OE transgenic lines are more susceptible to RDV and SRBSDV infection (A&E) Photographs of mock or RDV-infected (A) or SRBSDV-infected (E) WT and p2 OE transgenic rice lines. Scale bar, 10 cm. Photos were taken at 4 wpi. (B&F) Quantitative real-time PCR analysis of RDV S2 and S11 (B) or SRBSDV S7 and S10 (F) RNA accumulation in the indicated plants. Data were presented as mean ± SD (*n* = 8). (C&G) Immunoblot analysis of RDV P2 and Pns11 (C) or SRBSDV P7-2 and P10 (G) levels in the indicated plants. Actin was used as an internal control. (D&H) Disease incidence of rice plants infected with RDV (D) or SRBSDV (H) at 4 wpi and 8wpi. Data were shown as mean ± SD (*n* = 3). The significant differences were determined by the Student’s *t*-test (* *p* < 0.05; ** *p* < 0.01, *** *p* < 0.001). Figure S7. Performance of BPH on WT and brittle culm rice plants. (A&H) Photographs showing the distribution of BPH individuals on WT and *bc1* (A) or *bc13* (H) rice plants at 72 hpi. (B&I) Time-course analysis of the number of BPH individuals settled on WT and *bc1* (B) or *bc13* (I) rice plants at 6, 12, 24, 48, 72, 96, and 120 hpi. Data were shown as mean ± SD (*n* = 3). (C&J) Photographs of BPH oviposition on WT and *bc1* (C) or *bc13* (J) rice plants stained to visualize egg deposition. (D&K) Quantification of the number of eggs laid by BPH on WT and *bc1* (D) or *bc13* (K) rice plants. Data were shown as mean ± SD (*n* = 6). (E&L) Representative images of honeydew excretion spots produced by BPH feeding on WT and *bc1* (E) or *bc13* (L) rice plants. (F&M) Quantification of honeydew area on WT and *bc1* (F) or *bc13* (M) rice plants as a proxy for insect feeding activity. Data were shown as mean ± SD (*n* = 6). (G&N) Photographs of WT, *bc1* (G) or *bc13* (N) rice plants, and susceptible control TN1 rice plants before (0 day) and after (8 days) BPH inoculation. Scale bar, 5 cm. The significant differences were determined by the Student’s *t*-test (* *p* < 0.05; ** *p* < 0.01).

Supplementary Material 2.

Supplementary Material 3.

## Data Availability

All data generated or analyzed during this study are included in this published article and its supplementary information files.

## Abbreviations

Bc: Brittle culm
BPH: Brown planthopper
CESA: Cellulose synthase
CW: Cell wall
CWI: Cell wall integrity
dpi: Days post-inoculation
hpi: Hours post-inoculation
KEGG: Kyoto Encyclopedia of Genes and Genomes
OE: Overexpression
RDV: Rice dwarf virus
RGSV: Rice grassy stunt virus
RNA-seq: RNA sequencing
RT-qPCR: Reverse transcription-quantitative polymerase chain reaction
SRBSDV: Southern rice black-streaked dwarf virus
wpi: Weeks post-inoculation
WT: Wild type
