# Supplementary material for: A viral protein disrupts rice cell wall integrity and modulates interactions with viruses and insects
Source: Stress Biol. 2026 Feb 19;6(1):17. doi: 10.1007/s44154-026-00287-4 (PMC12921124; doi:10.1007/s44154-026-00287-4)
Supplement: Supplementary file 1 — Supplementary Material 1: Figure S1. Phenotype of WT and p2 OE rice plants. (A) Morphology of WT and p2 OE transgenic rice lines. Scale bar, 15 cm. (B) Tillering number comparison. Data were presented as mean ± SD (n = 10). (C) Productive ear number comparison. Data were presented as mean ± SD (n = 10). (D) 1000-grain weight comparison. Data were presented as mean ± SD (n = 10). (E) Statistical analysis of plant height in WT and p2 OE transgenic rice lines. Data were presented as mean ± SD (n = 10). (F) Grain shape comparison. Ten well-filled grains are presented in a column. Scale bar, 1 cm. (G) Comparison of grain length. Data were presented as mean ± SD (n = 100). (H) Comparison of grain width. Data were presented as mean ± SD (n = 100). The significant differences were determined by the Student’s t-test (*** p < 0.001). Figure S2. The length of cells and internodes of the WT and the p2 OE transgenic plants. (A) An image showing stems of the WT and the p2 OE#1 plants. Arrowheads indicate the stem nodes. I–IV, the first to the fourth internodes. The image was taken at 42-day post-planting. Scale bar, 10 cm. (B) The length of individual internodes relative to the total culm length. (C) Microscopic images showing the length of cells in the sheath of the WT and the three p2 OE transgenic lines. Scale bar, 300 µm. (D) Statistical analysis of cell length. Data were presented as mean ± SD (n = 100). The significant differences were determined by the Student’s t-test (** p < 0.01, *** p < 0.001). Figure S3. Staining of cellulose in leaf and stem sections. (A&C) Images showing cellulose contents in leaf sections from the WT and the p2 OE#1 plants (A) or mock and RGSV-infected plants (C). Cellulose in the leaf sections was stained with Calcofluor white. Scale bar, 50 μm. (B&D) Images showing cellulose contents in stem sections from the WT and the p2 OE#1 plants (B) or mock and RGSV-infected plants (D). Cellulose in the leaf sections was stained with Calcofluor white (E) St [file 44154_2026_287_MOESM1_ESM.docx]

**
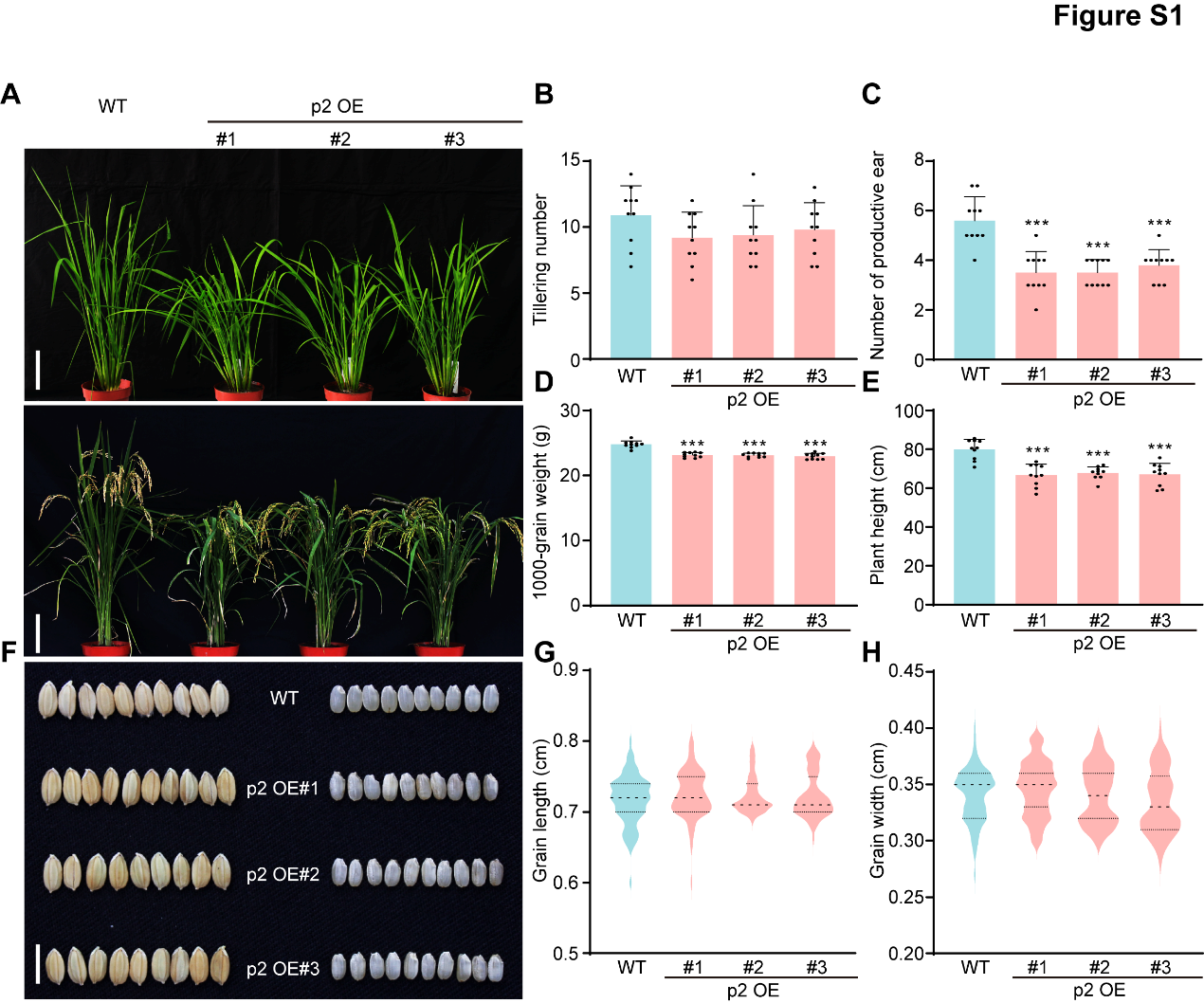
**

**Figure S1. Phenotype of WT and p2 OE rice plants.**

**(A)** Morphology of WT and p2 OE transgenic rice lines. Scale bar, 15 cm.
**(B)** Tillering number comparison. Data were presented as mean ± SD (*n* = 10).
**(C)** Productive ear number comparison. Data were presented as mean ± SD (*n* = 10).
**(D)** 1000-grain weight comparison. Data were presented as mean ± SD (*n* = 10).
**(E)** Statistical analysis of plant height in WT and p2 OE transgenic rice lines. Data were presented as mean ± SD (*n* = 10).
**(F)** Grain shape comparison. Ten well-filled grains are presented in a column. Scale bar, 1 cm.

**(G)** Comparison of grain length. Data were presented as mean ± SD (*n* = 100).

**(H)** Comparison of grain width. Data were presented as mean ± SD (*n* = 100).

The significant differences were determined by the Student’s *t*-test (*** *p* < 0.001).

**
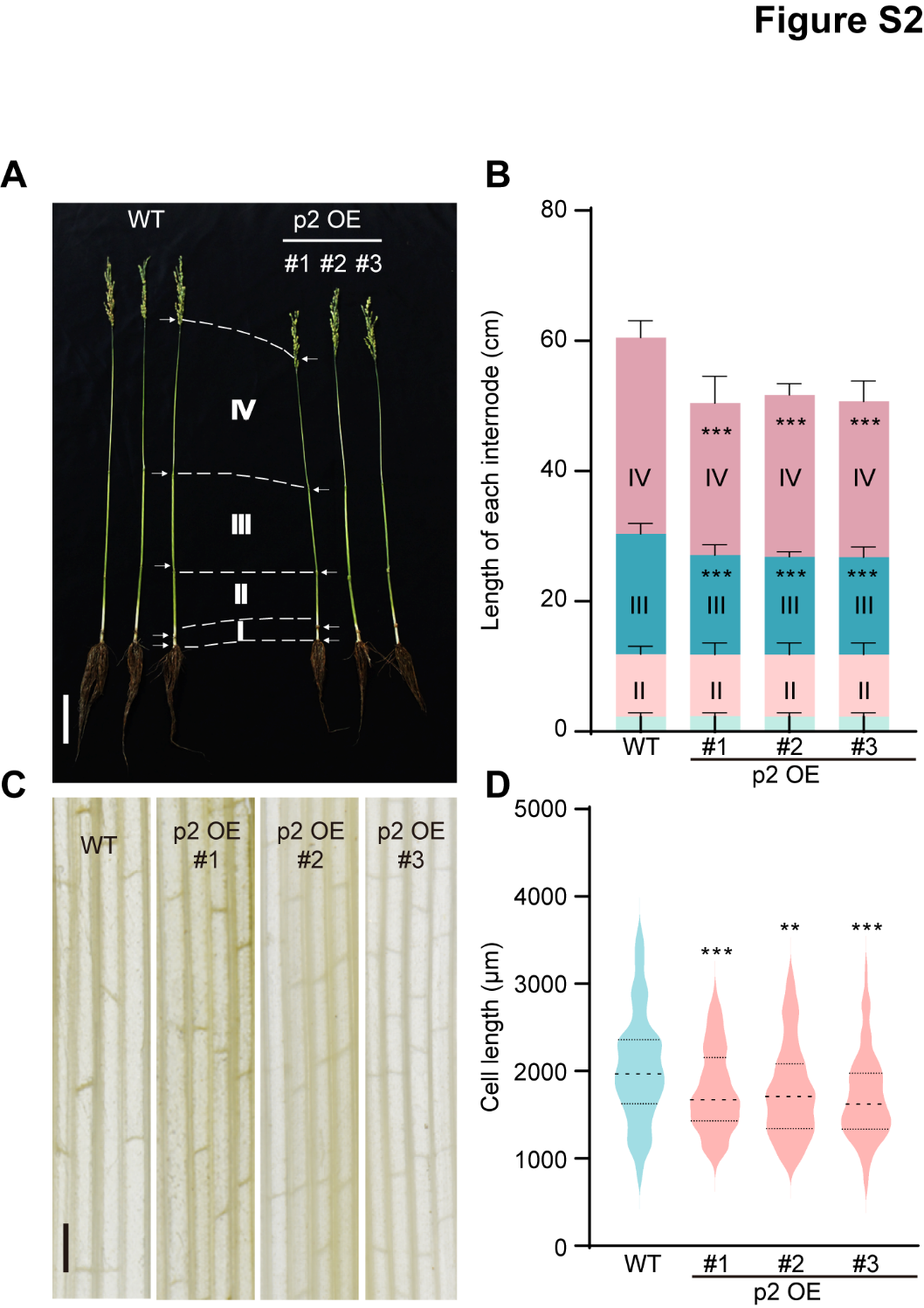
**

**Figure S2. The length of cells and internodes of the WT and the p2 OE transgenic plants.**

**(A)** An image showing stems of the WT and the p2 OE#1 plants. Arrowheads indicate the stem nodes. I–IV, the first to the fourth internodes. The image was taken at 42-day post-planting. Scale bar, 10 cm.
**(B)** The length of individual internodes relative to the total culm length.
**(C)** Microscopic images showing the length of cells in the sheath of the WT and the three p2 OE transgenic lines. Scale bar, 300 µm.
**(D)** Statistical analysis of cell length. Data were presented as mean ± SD (*n* = 100).

The significant differences were determined by the Student’s *t*-test (** *p* < 0.01, *** *p* < 0.001).


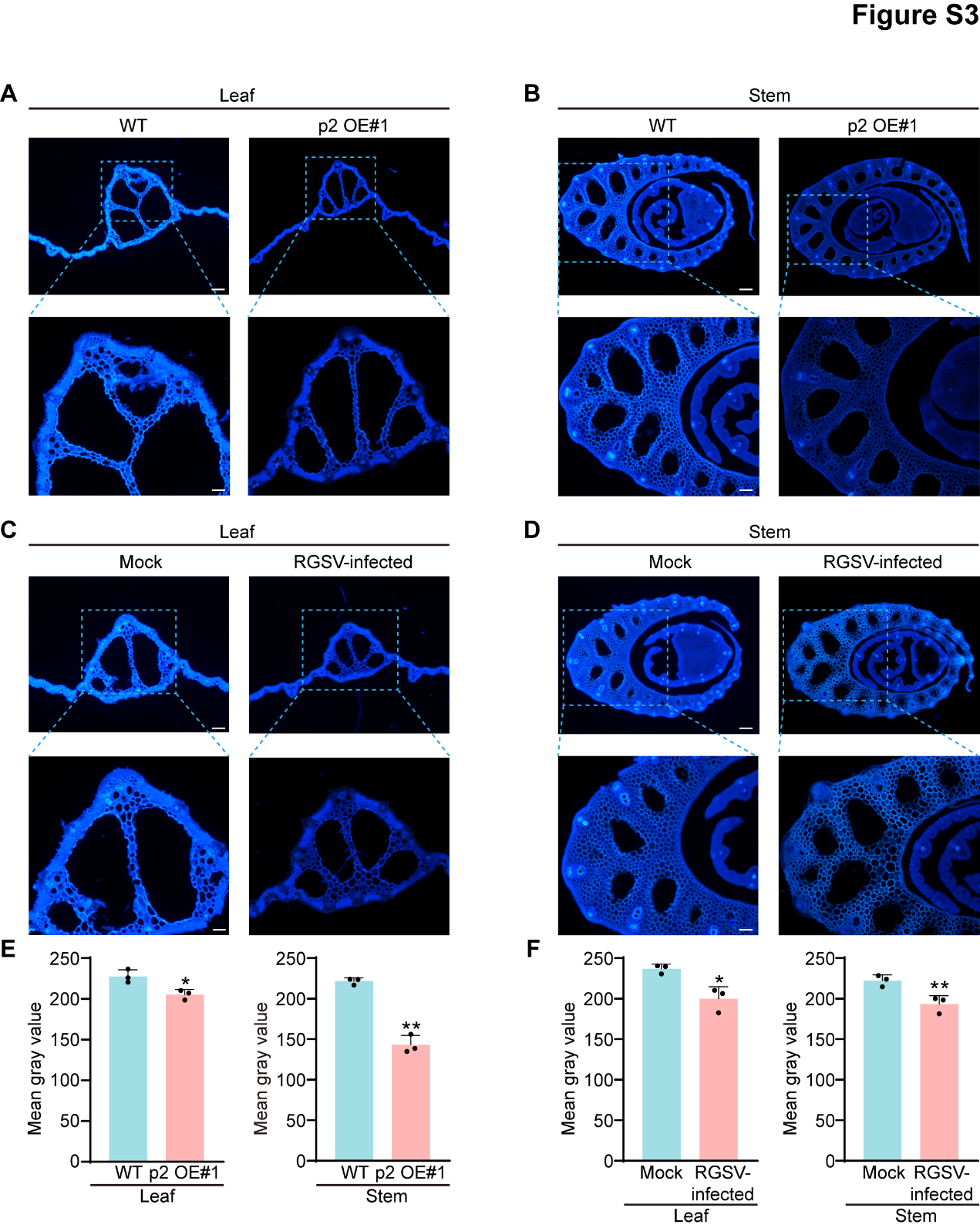


**Figure S3. Staining of cellulose in leaf and stem sections.**

**(A＆C)** Images showing cellulose contents in leaf sections from the WT and the p2 OE#1 plants (A) or mock and RGSV-infected plants (C). Cellulose in the leaf sections was stained with Calcofluor white. Scale bar, 50 μm.
**(B＆D)** Images showing cellulose contents in stem sections from the WT and the p2 OE#1 plants (B) or mock and RGSV-infected plants (D). Cellulose in the leaf sections was stained with Calcofluor white
**(E)** Statistical analysis of cellulose staining fluorescence intensity in leaves and stems of WT and p2 OE#1 plants. Data were shown as mean ± SD (*n* = 3).
**(F)** Statistical analysis of cellulose staining fluorescence intensity in leaves and stems of mock and RGSV-infected plants. Data were shown as mean ± SD (*n* = 3).

The significant differences were determined by the Student’s *t*-test (* *p* < 0.05; ** *p* < 0.01).

**
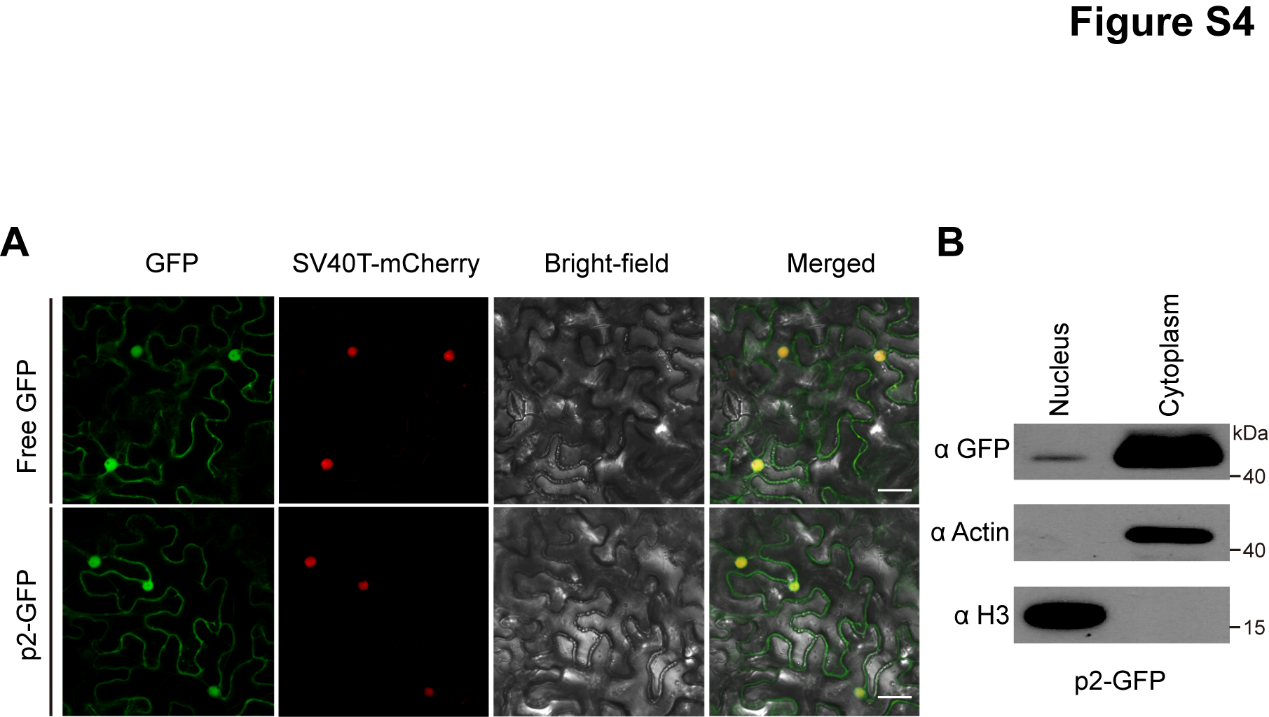
**

**Figure S4. Subcellular localization of p2.**

**(A)** Subcellular localization of RGSV p2 in *Nicotiana benthamiana* leaf cells. Scale bar, 10 µm.
**(B)** RGSV p2 was detected in the cytoplasmic protein fraction through Western blot assay. Actin and H3 in the fractions were also detected and used as the markers for cytoplasm and nuclear, respectively.

**
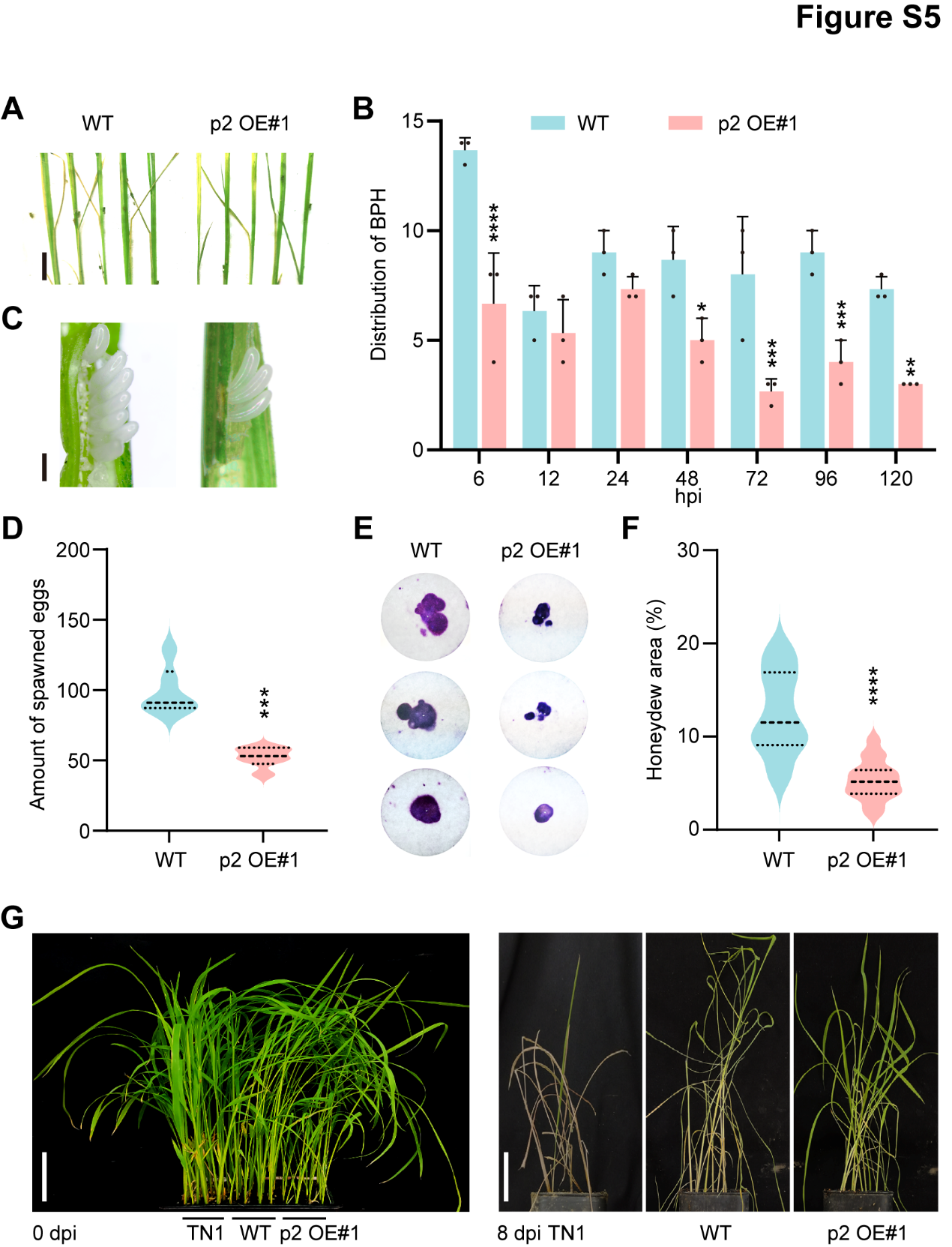
**

**Figure S5. Performance of BPH on WT and p2 OE rice plants.**

**(A)** Photographs showing the distribution of BPH individuals on WT and p2 OE rice plants at 72 hours post-inoculation (hpi).
**(B)** Time-course analysis of the number of BPH individuals settled on WT and p2 OE plants at 6, 12, 24, 48, 72, 96, and 120 hpi. Data were shown as mean ± SD (*n* = 3).
**(C)** Photographs of BPH oviposition on WT and p2 OE plants stained to visualize egg deposition.
**(D)** Quantification of the number of eggs laid by BPH on WT and p2 OE plants. Data were shown as mean ± SD (*n* = 6).
**(E)** Representative images of honeydew excretion spots produced by BPH feeding on WT and p2 OE plants.
**(F)** Quantification of honeydew area on WT and p2 OE plants as a proxy for insect feeding activity. Data were shown as mean ± SD (*n* = 15).
**(G)** Photographs of WT, p2 OE, and susceptible control TN1 rice plants before (0 day) and after (8 days) BPH inoculation. Scale bar, 5 cm.

The significant differences were determined by the Student’s *t*-test (* *p* < 0.05; ** *p* < 0.01, *** *p* < 0.001, **** *p* < 0.0001).


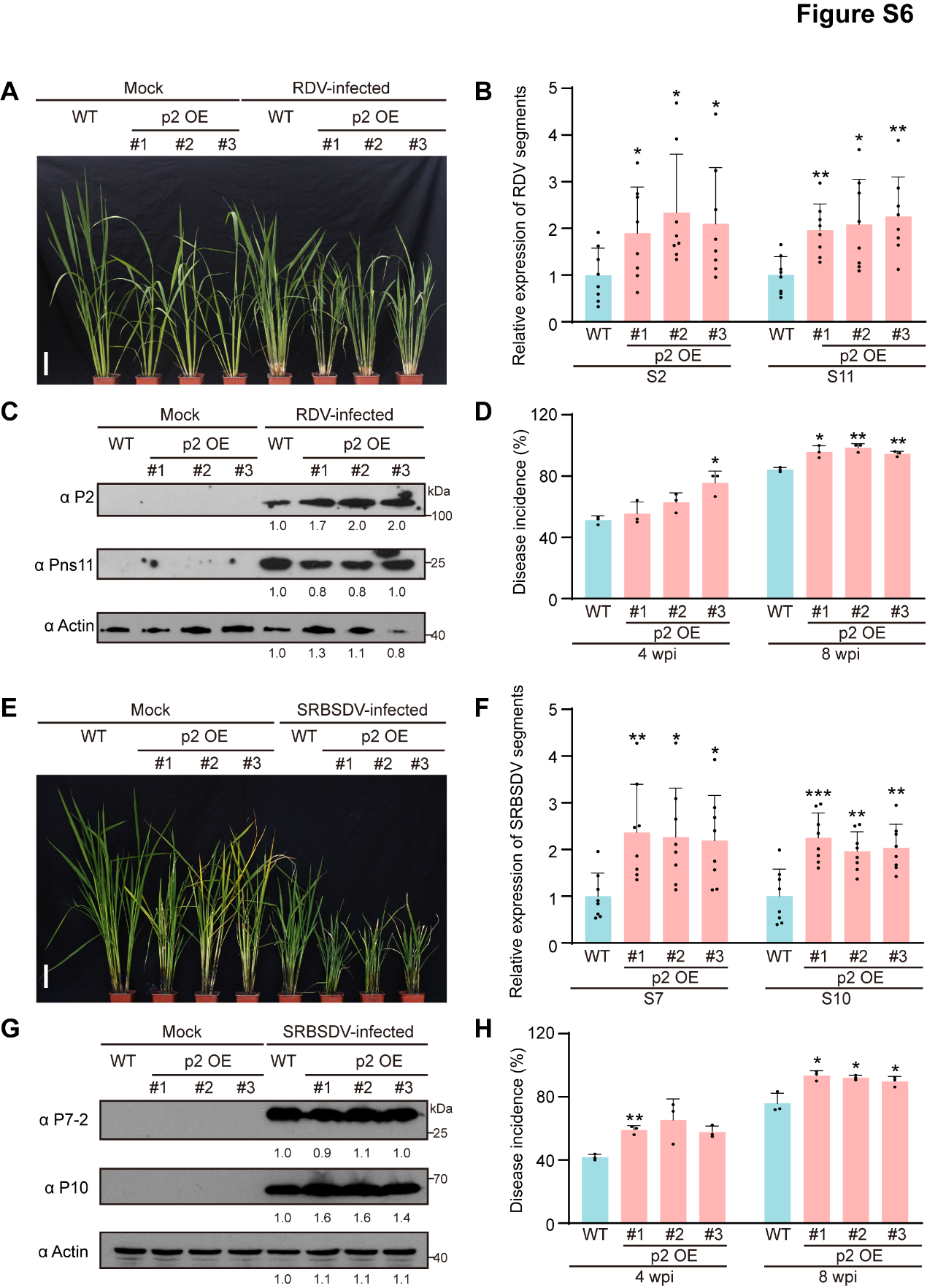


**Figure S6. The p2 OE transgenic lines are more susceptible to RDV and SRBSDV infection**

**(A＆E)** Photographs of mock or RDV-infected (A) or SRBSDV-infected (E) WT and p2 OE transgenic rice lines. Scale bar, 10 cm. Photos were taken at 4 wpi.
**(B＆F)** Quantitative real-time PCR analysis of RDV S2 and S11 (B) or SRBSDV S7 and S10 (F) RNA accumulation in the indicated plants. Data were presented as mean ± SD (*n* = 8).

**(C＆G)** Immunoblot analysis of RDV P2 and Pns11 (C) or SRBSDV P7-2 and P10 (G) levels in the indicated plants. Actin was used as an internal control.

**(D＆H)** Disease incidence of rice plants infected with RDV (D) or SRBSDV (H) at 4 wpi and 8wpi. Data were shown as mean ± SD (*n* = 3).

The significant differences were determined by the Student’s *t*-test (* *p* < 0.05; ** *p* < 0.01, ** *p* < 0.001).


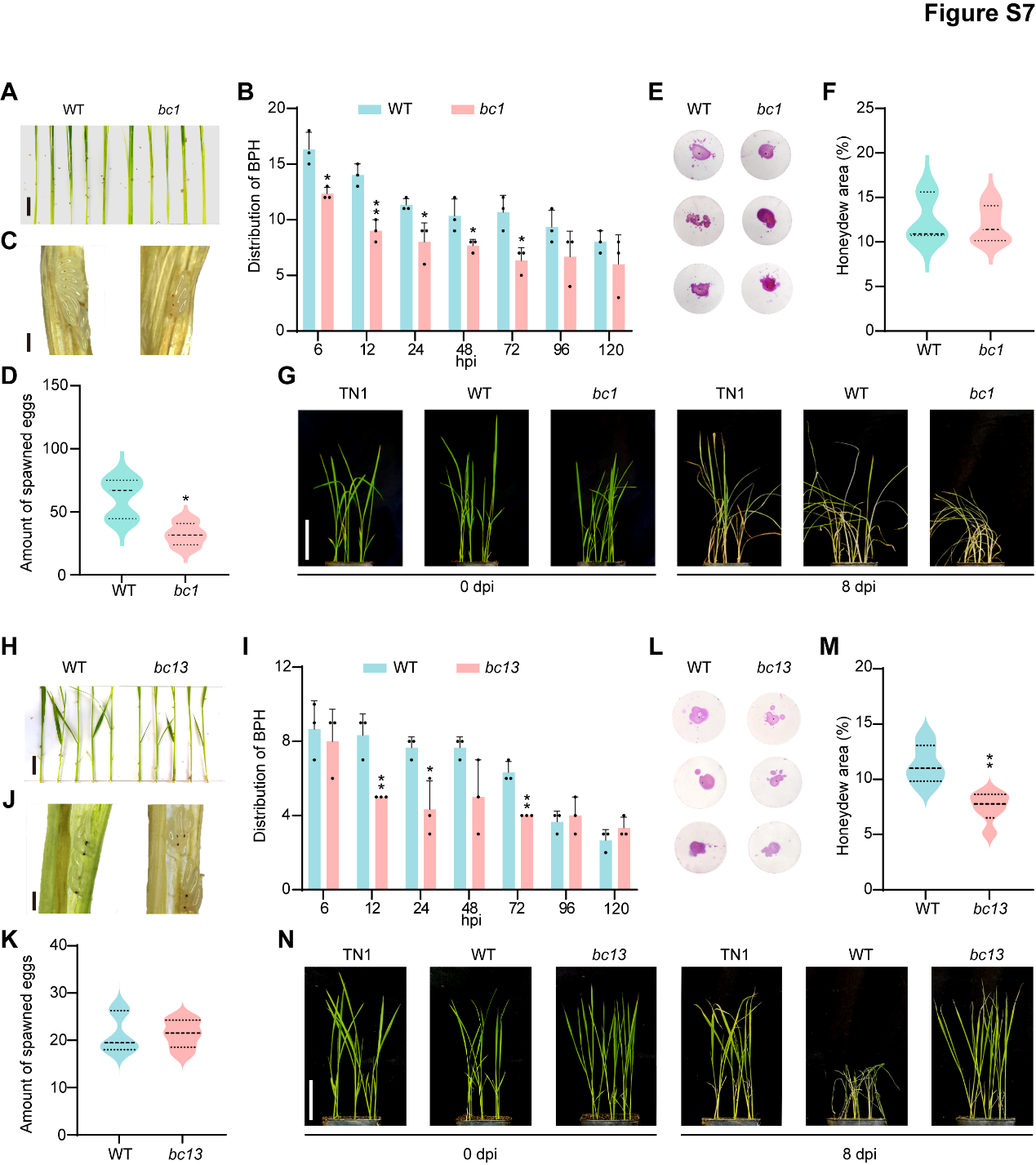


**Figure S7. Performance of BPH on WT and brittle culm rice plants.**

**(A＆H)** Photographs showing the distribution of BPH individuals on WT and *bc1* (A) or *bc13* (H) rice plants at 72 hpi.
**(B＆I)** Time-course analysis of the number of BPH individuals settled on WT and *bc1* (B) or *bc13* (I) rice plants at 6, 12, 24, 48, 72, 96, and 120 hpi. Data were shown as mean ± SD (*n* = 3).
**(C＆J)** Photographs of BPH oviposition on WT and *bc1* (C) or *bc13* (J) rice plants stained to visualize egg deposition.
**(D＆K)** Quantification of the number of eggs laid by BPH on WT and *bc1* (D) or *bc13* (K) rice plants. Data were shown as mean ± SD (*n* = 6).
**(E＆L)** Representative images of honeydew excretion spots produced by BPH feeding on WT and *bc1* (E) or *bc13* (L) rice plants.
**(F＆M)** Quantification of honeydew area on WT and *bc1* (F) or *bc13* (M) rice plants as a proxy for insect feeding activity. Data were shown as mean ± SD (*n* = 6).
**(G＆N)** Photographs of WT, *bc1* (G) or *bc13* (N) rice plants, and susceptible control TN1 rice plants before (0 day) and after (8 days) BPH inoculation. Scale bar, 5 cm.

The significant differences were determined by the Student’s *t*-test (* *p* < 0.05; ** *p* < 0.01).
